# Boron Nitride Nanosheets Functionalized with Fe_3_O_4_ and CoFe_2_O_4_ Magnetic Nanoparticles for Nanofiltration Applications

**DOI:** 10.1021/acsanm.3c02375

**Published:** 2023-06-22

**Authors:** Garret Dee, Olivia O’Donoghue, Aran Rafferty, Lee Gannon, Cormac McGuinness, Yurii K. Gun’ko

**Affiliations:** † School of Chemistry, University of Dublin, Trinity College, Dublin Dublin 2, Ireland; ‡ School of Physics University of Dublin, Trinity College, Dublin Dublin 2, Ireland

**Keywords:** boron nitride, nanosheets, magnetic nanoparticles, nanofiltration, water remediation

## Abstract

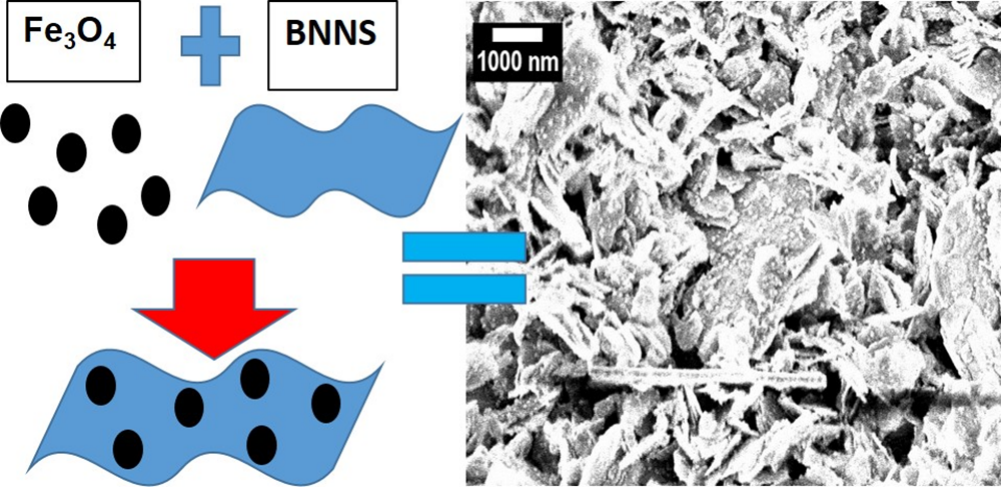

Nanofiltration (NF) is one of the emerging technologies that is very promising for water purification among many other applications. 2D boron nitride (BN) based nanomaterials are excellent building blocks for NF membranes. In our work, BN nanosheets (BNNS) have been functionalized with magnetic nanoparticles (MNPs) to form BNNS–MNP nanocomposites. It was found that the nanocomposites are stable with the MNPs giving very good coverage with both magnetite and cobalt ferrite MNPs and showing good attachment and stability to sonication. These nanocomposites have been tested for removal of methylene blue (MB) dye and MNPs from water. BNNS–magnetite nanocomposites showed higher removal efficiency of the MB from water than the corresponding pure BNNS, while the BNNS–cobalt ferrite removal efficiency was slightly less than the pure BNNS. The BNNS–cobalt ferrite material was regenerated by burning off the MB and recycled to show the recyclability of this material. The BNNS membranes were tested for filtration of 14 ± 4 nm magnetite MNPs and were found to capture 100% of the nanoparticles with no MNPs left in the filtrate. Thus, we have developed magnetic nanocomposite membranes, which have demonstrated great potential for water remediation. We believe that this research opens up promising ways for production of 2D nanocomposite materials with multiple applications.

## Introduction

Boron nitride (BN) is a very useful and attractive 2D nanomaterial for potential application in advanced nanofiltration.^1^ Hexagonal boron nitride, h-BN, is composed of 2D layered sheets of BN and is known as “white graphite”.^2^ It is colorless, transparent, electrically insulating, chemically stable, and mechanically strong. h-BN also has a high specific surface area and high thermal and chemical stability, with a high resistance to oxidation.^3^ These unique properties of h-BN have enabled the usage of this material in many applications including pollutant removal from water,^4,5^ as lubricants,^6^ and as super hydrophobic coatings.^7^ Boron nitride nanosheets (BNNS) can be made both by exfoliation of the bulk h-BN^8,9^ as a top down approach or by chemical synthesis as a bottom up approach.^10^

Magnetic nanoparticles (MNPs) have been extensively explored over many years due to their unique properties. These properties have found application in magnetic targeted drug delivery,^11^ magnetic sensing, magnetic resonance imaging (MRI) diagnostics,^12^ and magnetic heating in cancer hyperthermia therapy^13^ while also being studied for pollutant removal when combined with adsorption materials.^14^ MNPs can be prepared by numerous methods, which include coprecipitation,^15^ thermal decomposition,^16^ and thermal synthesis.^17^ Previously, a significant amount of work has been done in developing core@shell MNPs with the magnetic functionality at the core for catalysis applications.^18,19^ Another approach to introduce magnetic functionality is to coat the surface of another material with the magnetic material.

In recent years, boron nitride nanosheet–magnetic nanoparticle (BNNS–MNP) composites have been explored due to the vast number of potential applications that the combination of these two nanomaterials allow. BN coated with magnetite has already been reported for a variety of applications such as for toxic heavy metal ion removal from water,^20^ enhancing the thermal conductivity of an interfacial material when used in composite,^21^ as an electromagnetic wave-absorbing composite,^22^ and electronic packaging materials with excellent thermal stability.^23^ In all these cases, the coating of the BNNS with the magnetic material was an important factor in contributing to the added functionality and it was not a simple case of the mixing of the two materials.

This is a relatively recent area of research with not so many papers published on the topic compared with core@shell magnetic nanocomposites. One of the main challenges is the difficulty in attaching magnetic nanoparticles to the relatively inert BN surface. Previously, several groups have tried to functionalize BN prior to attachment of MNPs by various methods. One method reported to functionalize the BN surface was using a concentrated NaOH solution at 120 °C for 48 h to create hydroxide functional groups on the surface.^21^ Another approach involved reacting h-BN in the presence of di-*tert*-butyl peroxide, which decomposes at 120 °C by homolytic fission to produce oxygen radicals. These reactive species then attack the BN structure. Further modification finally produces hydroxyl-functionalized BNNS.^24^ These harsh methods have been understood to be necessary for attachment of other species to the BN surface. Other groups have reported attachment of iron oxide directly onto the BN surface without any modification of the surface. One example is with BN nanotubes (BNNT) coated with Fe_3_O_4_. The study indicated the likely sites that the Fe ions would bind to the bare BN surface through electrostatic interaction.^25^ Another report claimed to have created BNNS–Fe_3_O_4_ nanocomposites without functionalization of the BN surface by in situ coprecipitation of Fe_3_O_4_ with BNNS. However, the TEM (transmission electron microscopy) images in the paper are inconclusive and unclear regarding the attachment and coverage of magnetic material.^20^

In this work, we have developed a reproducible method to immobilize cobalt ferrite and iron oxide MNPs on BN nanosheets and produce BNNS–MNP nanocomposites with an excellent coverage of the nanosheets with MNPs without any functionalization prior to attachment of the MNPs. Then, we have used the BNNS–MNP 2D nanocomposites for the preparation of magnetic membranes and tested them in nanofiltration applications.

## Experimental Section

### Materials

Iron(III) chloride hexahydrate (reagent grade, ≥98%), iron(II) chloride tetrahydrate (p.a. ACS reagent), cobalt(II) chloride hexahydrate (reagent grade, ≥98%), ethylene glycol (anhydrous 98%), HMTA (reagent grade, 99%), methylene blue (reagent grade), ethanol (HPLC grade), and boron nitride (reagent grade) were supplied by Sigma-Aldrich. Sodium acetate (extra pure >99%) and Durapore 0.45 μm PVDF membrane were supplied by Merck. Ethylenediamine (>99%) was supplied by Merck-Schuchardt. Sodium hydroxide (reagent grade) was supplied by Fisher Scientific. MP H_2_O (Millipore water) was obtained using a Synergy 185 Millipore filtration system equipped with a 0.22 μM filter that was filtering distilled water. Technical grade solvents (>99%; ethanol, methanol, and acetone) supplied by Lennox were used for washing and storage.

### Instrumentation

The TEM used was the JEOL 2100 instrument operating at 200 kV. The SEM used was a Zeiss Ultra plus SEM, capable of an accelerating potential of 30–1 keV. The SEM was operated at between 15 and 2 keV to produce the images. EDX was done on the SEM with an Oxford Instruments 80mm^2^ XMAX EDX detector attached with the SEM operating at 15 keV. For the EDX analysis, the sample was placed on Lacey carbon TEM grids mounted on a holder in the SEM, and the SEM was focused onto an individual MNP-coated BN flake. The UV–vis spectra were obtained using an Agilent Cary 60 spectrophotometer with a range of 1100–190 nm and a quartz cuvette with a pathlength of 1 cm. pXRD was carried out using a Bruker, D2 Phaser 2nd generation, powder sample X-ray machine equipped with monochromatic high-intensity Cu Kα radiation (λ = 0.15406 nm). All the XRD data obtained was background-subtracted and was ran between 2θ angles of 15° to 85°. For obtaining magnetization measurements of the various dry products, an in-house assembled VSM was used at room temperature with field applied up to 1.1 T. The VSM was calibrated using a pure nickel sample of known mass. Nickel is a ferromagnetic material, which has a known magnetic moment of 55.4 A m^2^ kg^–1^ in an external field of 1 T at room temperature. FTIR spectra were obtained with a Perkin Elmer spectrum 100, equipped with a diamond window with an effective range of 4200–250 cm^–1^. BET surface area analysis was carried out using a Nova 2400e Surface Area Analyzer (Quantachrome, UK) with nitrogen gas as the adsorbate. The sample was degassed for 1 h at 200 °C under vacuum, prior to analysis. XPS measurements were obtained using an Omicron EA 125 Energy Analyzer with a monochromated Al Kα source at 1486.7 eV. High-resolution core-level component XPS scans were obtained with a pass energy of 20 eV, high magnification mode, and entrance and exit slits of 6 and 3 mm, respectively, giving an overall source and instrument resolution of 0.6 eV.

### Preparation of Boron Nitride Nanosheets (BNNS)

BNNS were prepared from bulk BN following previous work done in our group.^5^

Bulk BN powder (300 mg) and MP H_2_O (100 mL) were put in an RBF (150 mL). This solution was sonicated for 24 h (Wise Clean WUC-A03H operating at 40 kHz with 124 W output). This solution was used directly for transfer to ethylene glycol.

### Preparation of Sodium Ethoxide (EtONa)

Dry EtOH (85 mL, 1.47 mol) was degassed and put under argon in an RBF (250 mL). NaOH (5.87 g, 0.15 mol) was added and magnetically stirred under argon until dissolved. Molecular sieves (26.45 g, 300 mesh) were added, and the RBF was sealed under argon. The solution was left for 48 h. The liquid was separated from the molecular sieves under argon. The liquid was distilled (160 °C) under argon to remove excess EtOH, leaving a dry white powder of EtONa.

### Transfer of BNNS from Water to Ethylene Glycol

BNNS (300 mg in 100 mL of H_2_O) was added to ethylene glycol (120 mL) in an RBF (500 mL) and magnetically stirred. The water was distilled at 150 °C and collected. The distillation was stopped once 100 mL of water had been collected. The solution was cooled to RT and then sonicated for 1 h to disperse the nanosheets into the ethylene glycol.

### Preparation of Boron Nitride Nanosheets Iron Oxide Nanocomposites (BNNS–Fe_3_O_4_)

BNNS (100 mg, 4 mmol of BN) in ethylene glycol (40 mL) was degassed and put under argon in an RBF (100 mL). FeCl_3_·6H_2_O (0.16 g, 0.60 mmol) and ethylenediamine (0.40 mL, 6.0 mmol) were added and sonicated for 30 min under argon. EtONa (0.55 g, 8 mmol) in ethylene glycol (5.5 mL) was added to the solution. The solution was set up for mechanical stirring under argon and stirred for 30 min at RT to mix. The solution was refluxed (200 °C) for 16 h under argon. The solution was cooled to RT, and the particles were magnetically separated and washed with water (2 × 100 mL) and EtOH (2 × 100 mL) and stored in EtOH (100 mL).

### Preparation of Boron Nitride Nanosheets–Cobalt Ferrite Nanocomposites (BNNS–CoFe_2_O_4_)

BNNS (100 mg, 4 mmol of BN) in ethylene glycol (40 mL) was put in an RBF (100 mL). FeCl_3_·6H_2_O (0.109 g, 0.40 mmol), CoCl_2_·6H_2_O (0.048 g, 0.3 mmol), and ethylenediamine (0.40 mL, 6.0 mmol) were added and sonicated for 30 min in open air. EtONa (0.55 g, 8 mmol) in ethylene glycol (5.5 mL) was added to the solution. The solution was set up for mechanical stirring and stirred for 30 min at RT to mix. The solution was refluxed (200 °C) for 16 h in open air. The solution was cooled to RT, and the particles were magnetically separated and washed with water (2 × 100 mL) and EtOH (2 × 100 mL) and stored in EtOH (100 mL).

### Preparation of Boron Nitride Nanosheets–Magnetic Nanoparticle Nanocomposite Membranes

A solution of known concentration of the material was sonicated for 2 h to disperse the material fully. The solution was filtered on a PVDF 0.45 μM filter on fritted glass apparatus to create the membrane. The membrane was used for filtration immediately after being created with the PVDF membrane in place on the fritted glass apparatus, before it had dried out. For one of these membranes, it was allowed to dry and the mass and thickness of the membrane were measured.

### Testing of the Membrane for Extraction of Dye

A freshmembrane was produced from 40 mg of the desired material (BNNS, BNNS–Fe_3_O_4_, or BNNS–CoFe_2_O_4_). A solution of MB (20 mL, 21.9 μM) was filtered through the membrane, the filtrate was collected, and a UV–vis spectrum was taken. This was repeated with equal amounts of solution until the membrane became saturated.

### Testing the BNNS–CoFe_2_O_4_ for Regeneration and Recycling

A used BNNS–CoFe_2_O_4_ membrane was washed with MeOH and H_2_O until the filtrate ran clear. The membrane was sonicated in a minimum amount of acetone, and the PVDF support was removed. The material was magnetically extracted from the acetone. Then, the material was placed in a furnace at >400 ° C. The material was sonicated back in to solution in MP H_2_O (100 mL). This solution containing the material was then used to create a fresh membrane and the experiment was repeated.

### Testing of the Membrane for Magnetic Nanoparticle Filtration

A fresh membrane was made from 40 mg of the desired material (BNNS, BNNS–Fe_3_O_4_ or BNNS–CoFe_2_O_4_). A solution (20 mL) of freshly sonicated MNPs at a concentration of 0.2 mg/mL was filtered. The filtrate was collected and left on a magnet overnight to see if any MNPs were extracted from the filtrate.

### Synthesis of Fe_3_O_4_ (Polyol)

FeCl_3_·6H_2_O (3.50 g, 12.9 mmol) was mixed in ethylene glycol (50 mL) and mechanically stirred until dissolved. Separately, sodium acetate (3.54 g, 43.1 mmol) was added to ethylene glycol (50 mL) and stirred until dissolved. The two solutions were mixed and set up for heating with mechanical stirring. The solution was refluxed (BP EG is ∼198 °C) for 20 h under argon. The solution was cooled to RT, and the particles were extracted using a permanent neodymium magnet. The product was washed with MP H_2_O (100 mL), MeOH (2 × 100 mL), and acetone (100 mL). The final product was stored in EtOH for further use.

### Synthesis of CoFe_2_O_4_ (Polyol)

FeCl_3_·6H_2_O (2.30 g, 8.5 mmol) and CoCl_2_·6H_2_O (0.86 g, 4.3 mmol) were mixed in ethylene glycol (50 mL) and mechanically stirred until dissolved. Separately, sodium acetate (3.56 g, 43.4 mmol) was added to ethylene glycol (50 mL) and stirred until dissolved. The two solutions were mixed and set up for heating with mechanical stirring. The solution was refluxed (BP EG is ∼198 °C) for 20 h with the condenser open to air. The solution was cooled to RT, and the particles were extracted using a permanent neodymium magnet. The product was washed with MP H_2_O (100 mL), MeOH (2 × 100 mL), and acetone (100 mL). The final product was stored in EtOH for further use.

### Synthesis of Fe_3_O_4_ (Coprecipitation)

NH_4_Cl (0.03 g, 0.56 mmol) was dissolved in MP H_2_O (50 mL), and the solution was degassed and put under with argon. Stock (25%) ammonia solution (4 mL, 58 mmol) was added, and the solution was set up for mechanical stirring under argon and heated to 90 °C. Separately FeCl_2_·4H_2_O (0.09 g, 0.43 mmol) and FeCl_3_·6H_2_O (0.23 g, 0.86 mmol) were dissolved in degassed MP H_2_O (20 mL) under argon. The iron solution was added dropwise to the ammonia solution. The solution was left stirring for 2 h under argon at 90 °C. After this, the solution was allowed to cool to RT and then the product was magnetically extracted. The product was washed with MP H_2_O (100 mL), MeOH (2 × 100 mL), and acetone (100 mL). The final product was stored in MP H_2_O (500 mL) for further use.

## Results and Discussion

Initially, our work was focused on the development of a method to coat BNNS with magnetic iron oxide to achieve high surface coverage and produce BNNS–Fe_3_O_4_ nanocomposites. The process involved the use of BNNS, which have been exfoliated in water from bulk boron nitride similar to previously reported procedures.^9^ The BNNS solution was then transferred to a polyol solvent for the further coating with MNPs. A variety of different modifications was tried to achieve a maximal coating on the BNNS. These included changing the amounts of precursor and changing the base used in the polyol synthesis. The method was also found to be effective both with a magnetite (Fe_3_O_4_) and a cobalt ferrite (CoFe_2_O_4_) MNPs coating with some minor differences. CoFe_2_O_4_ is more chemically and thermally stable than Fe_3_O_4_ as it can be heated up to 1000 °C^26^ without loss of its magnetism. For the creation of a membrane that can be recycled *in situ* by magnetic induction heating to burn off collected organic residue, CoFe_2_O_4_ may be a more promising option and this was the rationale behind following that line of research.

### Preparation of BNNS

BNNS has been made from bulk BN by sonication in water for 24 h as described in the experimental section.

**Figure 1:**
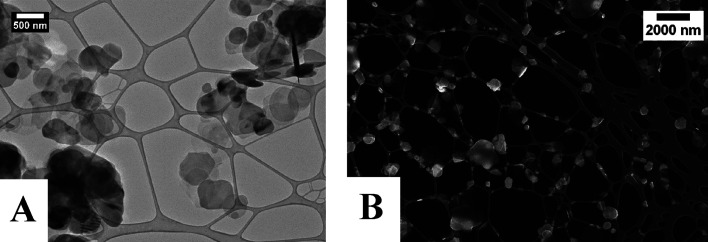
(A) TEM and (B) SEM images of BNNS on a Lacey carbon TEM grid.

TEM and SEM images of the BNNS nanosheets are presented here (Figure 1 and Figures S1 and S2). The sample consists mainly of individual nanosheets with some aggregation due to sample drying for the TEM characterization. SEM images of BNNS on the TEM grid show similar outcomes with the individual sheets visible on the TEM grid. As can be seen, the individual flakes vary in size greatly with length and width dimensions ranging from 50 to 1000 nm. In addition, some flakes show packing where flakes are layering on each other. This would be due to the drying effects when preparing the sample for electron microscopy image.

X-ray Diffraction (XRD) of the BNNS gave an exact match for h-BN in the COD database (COD 201-6171) with the major peaks at 2θ and corresponding planes of 26.7° (002), 41.6° (010), 43.8° (011), 50.1° (012), 55.0° (004), 71.3° (014), 75.9° (110), and 82.2° (112) as can be seen (Figure S3). There was no difference to an XRD of the powder BN.

Fourier transform infrared (FTIR) spectra of the BNNS (Figure S4) showed strong peaks at 740 and 1300 cm^–1^ where previous work has attributed these peaks to the out-of-plane vibration of B–N–B and the in-plane vibration of B–N–B, respectively.^26^ These peaks were also in the powder BN prior to exfoliation as would be expected.

X-ray photoemission spectroscopy (XPS) survey scan of the BNNS samples (Figure S5) shows peaks for the B1s and N1s core levels at 191.01 and 398.41 eV, respectively, when calibrated to the adventitious C1s peak, which are in good agreement with previously reported values for B and N.^24^ The spectrum also shows traces of C and O from adsorbed contaminants, which are common impurity in XPS samples exposed to the atmosphere and can be used for calibration of the peak positions.^27^ A high-resolution scan of the B1s region shows the raw peak to be a combination of two separate peaks when deconvoluted with a constraint to keep the full width at half-maximum (FWHM) the same for the two peaks (Figure S6). According to previous reports, these peaks can be assigned to the B–N for the major peak at a binding energy of 191.01 eV and B–O for the minor peak at 192.00 eV, with the O possibly coming from both terminal oxygens^28^ and absorbed O on the BN sheets^29^ from O_2_ and H_2_O.

These initial characterizations for the BNNS will be used for reference as we discuss the MNP-coated BNNS in the following sections.

### Preparation of BNNS–Fe_3_O_4_ Nanocomposites

**Scheme 1:**
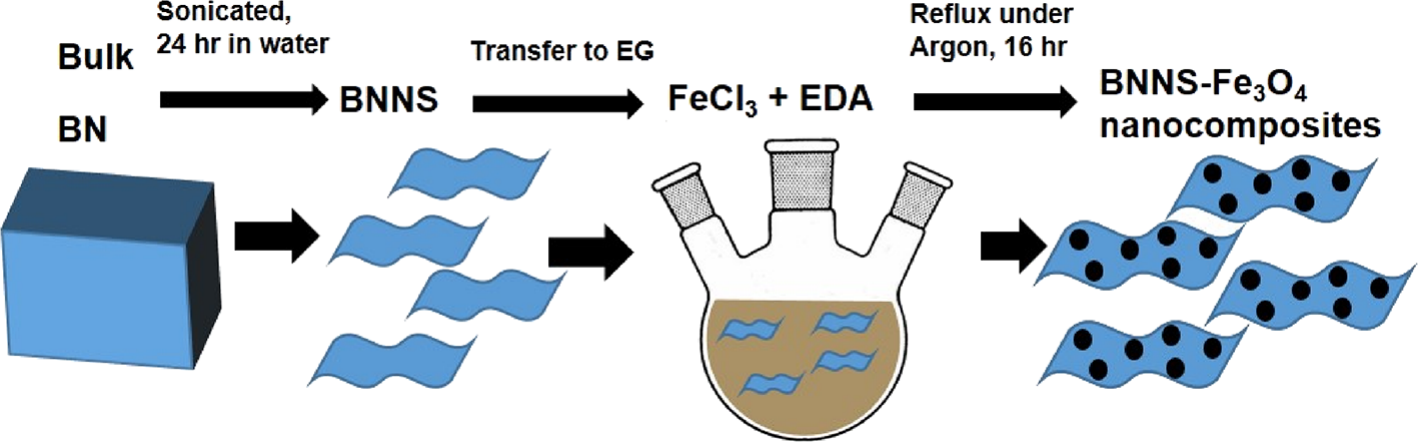
Synthetic Process to Form the BNNS–Fe_3_O_4_ Nanocomposites

BNNS–Fe_3_O_4_ nanocomposites have been prepared according to a procedure developed by us, which is described in detail in the experimental section. A simplified outline of the process is shown (Scheme 1). The procedure involves transfer of the exfoliated BNNS to ethylene glycol so that a polyol-type reaction can be performed in situ with the BNNS. A molar ratio for BN:Fe_3_O_4_ of 1:0.05 was chosen to make sure that there was not an excess of Fe_3_O_4_. This ratio gives one Fe atom per six BN rings. This method was found to give well-coated BNNS. This synthesis was performed several times and was found to be highly reproducible. The BNNS–Fe_3_O_4_ nanocomposites and the corresponding membranes were characterized by SEM, EDX, TEM, FTIR, vibrating-sample magnetometer (VSM), XRD, XPS, and BET.

**Figure 2:**
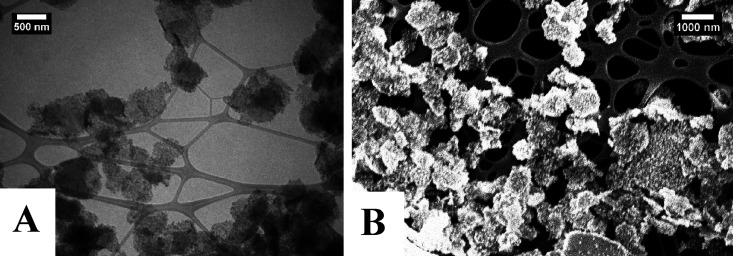
(A) TEM and (B) SEM image of the Fe_3_O_4_ coated BNNS.

The TEM and SEM images (Figure 2 and Figure S7) of the BNNS–Fe_3_O_4_ nanocomposites show BN nanosheets well coated with MNPs. It should be noted that no separate MNPs were found in the images with all the MNP on the surface of the BNNS. Some BNNSs were more heavily coated than others were though but in general, there was consistent and uniform coverage of the BN flakes across the sample. The iron oxide coverage consisted mainly of magnetic nanoparticles of approximately 6 ± 3 nm in diameter but with some larger particles too. Particle size distributions were calculated from 100 particles in the TEM images using ImageJ software. A size distribution chart is shown (Figure S8). These MNPs were on the BNNS, which had varying dimensions of length and width from 100 to 1000 nm.

During SEM analysis, an energy-dispersive X-ray (EDX) detector attached to the SEM was used to do EDX analysis of the BNNS–Fe_3_O_4_ sample to gain insights into the ratio of elements in the sample and to map the position of the elements. Elemental maps for the BNNS–Fe_3_O_4_ sample are shown (Figure S9). As can be seen in the mapping, Fe and O are evenly distributed giving a good coating of the BN sheet. This confirms the SEM and TEM results above (Figure 2), showing that the BNNS is well coated with the MNPs. For EDX quantification, a thick layer of the sample was deposited onto carbon stubs and a wide field scan was taken, thus reducing any differences in local coverage. From the EDX quantification results, the atomic percentages of the Fe:O:B:N were found to be 1.91:29.43:34.37:34.29. The ratio of B:N was approximately 1:1 as expected while the ratio for Fe:O was higher than expected. This is most likely because of bound H_2_O and terminal oxygen atoms on the surface of the BN and iron oxide. It could also be due to the partial oxidation of the Fe_3_O_4_ to γ-Fe_2_O_3_, which is still magnetic and so the BN-MNP composite will not lose the magnetic functionality.^30^ The ratio of Fe:N was 1.91:34.29 giving an atomic ratio of 0.056. This was higher than the expected value atomic ratio of 1:0.02, indicating that some of the BN may have been washed away with the magnetic extraction washing steps.

FTIR analysis of the samples (Figure S10) showed peaks at 552 cm^–1^ corresponding to the Fe–O stretch of the iron oxide. The peaks at 740 and 1300 cm^–1^ have both shifted slightly to 790 and 1350 cm^–1^, respectively, as would be expected with the iron oxide interacting with the flakes of BN. The observed almost complete coverage would be expected to produce some change in the BN peaks. These shifts indicate new interactions resulting in changes in the out-of-plane and the in-plane vibration. Previous studies have reported the binding of iron oxides^20^ and Fe ions^25^ to the surface of BN. In the Fe ion study, the group looked at how Fe ions interacted with the surface of BN and found that the optimal binding site was at the center of the BN ring. They proposed the formation of borazine–metal complex bonds,^25^ which would result in the changes in the IR spectra of the BN similar to what is observed here. As the BN was sonicated with the Fe ions prior to the reaction, it is likely that this enabled the formation of these bonds. It is possible that as the Fe ions were in place as seeds for the growth of iron oxide, this then gives the excellent coverage seen when the reaction took place. The study used DFT calculations to show the optimized binding of iron oxide with the BN sheet with a view for increased adsorption of contaminants.^20^

In the XPS survey spectrum of the sample (Figure S5), we can see the peaks for B1s, N1s, Fe2p, and O1s, indicating the elemental composition of the sample, with a peak for adventitious C1s due to the presence of small amounts of impurities after exposure to the atmosphere, which was used for calibration.^27^ In the high-resolution B1s peak XPS scan (Figure S11A) of the BNNS–Fe_3_O_4_ sample compared with the BNNS sample, we can see a clear difference in the raw data shape and peak position. Deconvolution of the B1s peak of the BNNS–Fe_3_O_4_ sample (Figure S11B) shows it to be a combination of 3 peaks, with the FWHM constrained to be the same for the 3 deconvoluted peaks. We see a small shift to a lower binding energy compared with the pure BNNS for the B–N with a peak position of 190.89 eV and B–O with a 191.95 eV. The emergence of a small new peak at a higher binding energy of 193.2 eV can be attributed to the formation of a small number of B–Fe bonds. The FWHM here is reduced compared with the pure BNNS (1.22 compared with 1.29), indicating less chemical disorder of the B sites here. This indicates that as the MNPs bind to the BNNS with the proposed formation of B–Fe bonds, the electron density moves from the B to the Fe, while there is a concurrent movement of electron density on to the rest of the sheet, possibly due to back donation from the ferrite lattice to the BN sheet. The XPS analysis indicates that there is a complex interaction going on between the MNPs and the BN sheet.

**Figure 3:**
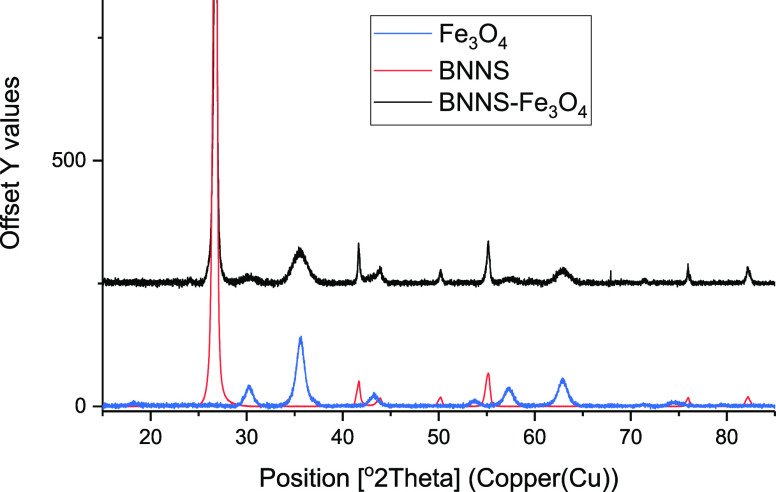
XRD patterns of BNNS–Fe_3_O_4_ shown to be a combination of h-BN and Fe_3_O_4_.

XRD analysis showed that the BNNS–Fe_3_O_4_ had a combination of the patterns of h-BN and Fe_3_O_4_ made previously and matched to the COD database, (COD 201-6171) and (COD 722-8111), respectively, as shown in Figure 3. It should be noted that these patterns had to be adjusted for count intensities but not peak positions. A phase quantification analysis (Figure S12) of the XRD result gave boron nitride as 98.8% of the sample with Fe_3_O_4_ as 1.2%. The phase quantification method is a percent mass of the material analysis. The major peaks at 2θ and corresponding planes have been assigned for the BN (blue) of 26.7° (002), 41.6° (010), 43.8° (011), 50.1° (012), 55.0° (004), 71.3° (014), 75.9° (110), and 82.2° (112) and the Fe_3_O_4_ (green) of 30.2° (022), 35.6° (113), 37.3° (222), 57.2° (115), and 63.0° (044).

VSM analysis gave a magnetization value of 13 A m^2^ kg^–1^ at 1 T. There was no residual magnetism when the field was zero T, and the shape of the magnetization curve indicated superparamagnetic behavior of the nanocomposites as there was no hysteresis loop and the magnetization has not saturated at 1 T, as can be seen in Figure S13.

Some variations to the procedure were done in order to optimize the coating. Instead of using EtONa as the base, NaOH was used instead. This gave good coverage too but slightly lower than with EtONa as can be seen in (Figure S14A). Another variation that was tried was doing the synthesis without ethylenediamine (EDA). This gave an outcome of larger particles on the surface of the BNNS as can be seen in Figure S14B with some areas of the BNNS that were not fully covered. These particles were approximately 40 ± 10 nm in diameter with very few of the smaller particles when using EDA. These MNPs were on the BNNS of varying dimensions with lengths and widths from 100 to 1000 nm as observed in the TEM images. Using EDA in the reaction gave more uniform coverage overall. These results showed that the use of EtONa and EDA in the synthesis gave the best results. Other variations could be carried out, such as changing the base used and the surface-active agents to control the particle growth or anisotropy, which we will plan to do in future work to further optimize these procedures.

### Preparation of BNNS–CoFe_2_O_4_ Nanocomposites

BNNS–CoFe_2_O_4_ nanocomposites were prepared by a similar procedure used to produce the BNNS–Fe_3_O_4_. The synthesis involved the transfer of the exfoliated BNNS to ethylene glycol for a polyol-type of reaction to take place to form CoFe_2_O_4_ MNPs with the BNNS *in situ*, enabling the formation of MNPs on the surface of the BNNS. The BNNS–CoFe_2_O_4_ nanocomposites were characterized by SEM, TEM, FTIR, VSM, EDX, BET, XPS, and XRD techniques.

**Figure 4:**
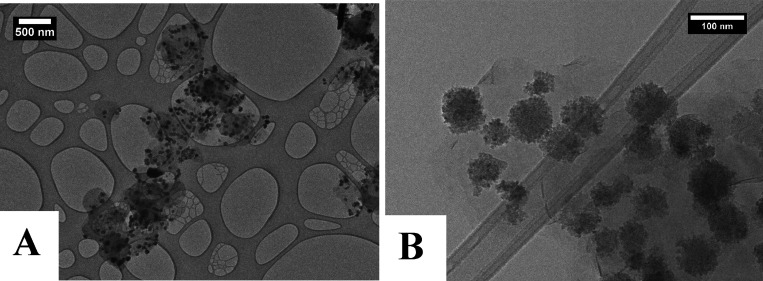
(A, B) TEM images of BNNS–CoFe_2_O_4_ showing larger CoFe_2_O_4_ particles on the BNNS surface.

A TEM image (Figure 4) of the BNNS–CoFe_2_O_4_ showed that the BNNS was coated with CoFe_2_O_4_ particles but not as uniformly as with the Fe_3_O_4_. As can be seen, larger CoFe_2_O_4_ particles had formed on the surface of the BNNS of approximately 60 ± 10 nm in diameter. Particle size distributions for the MNPs were calculated from 100 particles in the TEM images using ImageJ software. A size distribution chart is shown (Figure S15). These MNPs were on BN nanosheets of varying lengths and widths, from 100 to 1000 nm. Although the coverage was not as good as with the Fe_3_O_4_, there were no separate CoFe_2_O_4_ particles in the images. This indicated that all the particles had formed on the BNNS only.

EDX analysis was also performed on the BNNS–CoFe_2_O_4_ sample during SEM to gain insights into the ratio of elements in the sample and to map the elements on the surface of the BN sheets. Elemental maps for the BNNS–Fe_2_O_4_ sample are shown (Figure S16). As can be seen in the mapping images, Fe and Co are found to evenly coat the BN sheet, which is in line with the SEM and TEM results (Figure 4). From the EDX quantification for the atomic percentages of the Fe:Co:O:B:N were found to be 3.46:1.53:15.83:39.47:39.70. The ratio of B:N was 1:1 as expected while the ratio of Co:Fe was 1:2.3, which was close to the expected value of 1:2. The ratio of the metal ions to O (Fe + Co:O) was 1:3.2, which was higher than the theoretical expected value of 1:1.3. This again would indicate the presence of some adsorbed H_2_O molecules on the surface of the BN and MNPs. Finally, the ratio of Co + Fe:N was 1:0.13, which was higher than expected, indicating that some of the BN may have been washed away with the magnetic extraction procedures.

FTIR spectra (Figure S17) have shown bands at 563 cm^–1^ corresponding to the metal–oxygen stretch of the CoFe_2_O_4_. The peaks at 740 and 1300 cm^–1^ have both shifted slightly to 770 and 1340 cm^–1^, respectively. This shift was not as much for the BNNS–Fe_3_O_4_ corresponding to there being not as much coverage but indicating an interaction of the CoFe_2_O_4_ with the BNNS.

VSM analysis (Figure S18) gave a magnetization value of 17 A m^2^ kg^–1^ at 1 T. There was no coercivity or residual magnetization when the field was zero T. This indicates superparamagnetic behavior as there was no residual magnetization.^26^

In our XPS survey spectrum of the sample (Figure S5), we can see the peaks for B1s, N1s, Fe2p, Co2p, and O1s, indicating the elemental composition of the sample, with a peak for adventitious C1s as before, which has been used for calibration. Comparing the high-resolution B1s peak XPS scan (Figure S19A) of the BNNS–CoFe_2_O_4_ sample with that of the BNNS sample, we can see a clear difference in the raw data peak shape, with a noticeable broadening and an extended shoulder to higher binding energy. Deconvolution of the B1s peak of the BNNS–CoFe_2_O_4_ sample (Figure S19B) shows it to be a combination of 4 peaks, with the FWHM constrained to be the same for the 4 deconvoluted peaks. The FWHM here is increased compared with that of the pure BNNS (1.59 compared with 1.29), indicating more chemical disorder of the B sites here. We see a small shift to a lower binding energy compared with the pure BNNS for the B–N with a peak position of 190.95 eV, with no change for the B–O peak at 192.00 eV binding energy. The emergence of 2 small new peaks at a higher binding energies of 193.23 and 193.68 eV can be attributed to the formation of a small number of B–Fe and B–Co bonds, respectively, with the electron density moving from the B to the metal centers. There is also a reduction in the intensity of the B–O peak, which can be rationalized by the fact that the BNNS–CoFe_2_O_4_ was heated to 400 °C before the XPS analysis. This would have burned off any bound H_2_O. The broadening of the peak implies that a more complex chemical environment has been introduced with the addition of these MNPs to the BN sheets.

**Figure 5:**
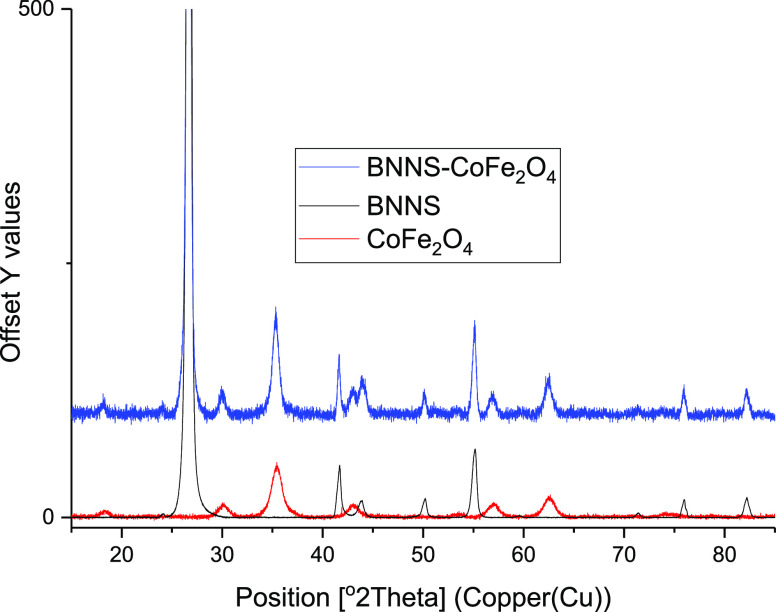
XRD patterns of BNNS–CoFe_2_O_4_ shown to be a combination of h-BN and CoFe_2_O_4_.

XRD analysis showed that the BNNS–COFe_2_O_4_ was a combination of the h-BN and CoFe_2_O_4_ patterns and matched the COD database (COD 201-6171) and (COD 153-3164), respectively, as shown in Figure 5. The peaks were adjusted for count intensities but not peak positions because without adjustment, the peaks for BNNS–CoFe_2_O_4_ would not be visible as its counts were much lower than the other materials were. A phase quantification analysis (Figure S20) of the XRD result gave boron nitride as 98.8% and CoFe_2_O_4_ as 1.2% of the sample. The major peaks at 2θ with corresponding planes have been assigned for the BN (blue) of 26.7° (002), 41.6° (010), 43.8° (011), 50.1° (012), 55.0° (004), 71.3° (014), 75.9° (110), and 82.2° (112) and the CoFe_2_O_4_ (green) of 18.3° (111), 30.2° (022), 35.6° (113), 37.3° (222), 57.2° (115), and 63.0° (044).

In order to optimize the synthesis of this material, we tried to use different ratios of BNNS to CoFe_2_O_4_. The initial sample had a molar ratio for BN:CoFe_2_O_4_ of 1:0.05. Samples were also made having ratios of 1:0.1 and 1:0.01 to see the effect on coverage and magnetic properties. As expected, the coverage of the MNPs on the BNNS was dependent of the ratio with the larger molar ratio giving high coverage and the lower ratio giving less coverage. The effect of this can be seen in the TEM images (Figure S21) where there is high coverage with the 0.1 ratio with some separate individual MNPs in the images. For the other two ratios, there is a decreasing level of coverage and no separate MNPs were observed in the images. The size of the MNPs on the surface remained constant with the changing molar ratio at approximately 40 ± 10 nm as can be seen in the images. The BNNSs were of similar sizes from 100 to 1000 nm in diameter in all the samples.

The magnetic properties were also dependent on the molar ratio of the non-magnetic BNNS and the magnetic CoFe_2_O_4_ with the magnetization being 27, 13, and 6 A m^2^ kg^–1^ for the 0.1, 0.05, and 0.01 ratios, respectively. The results of these observations are summarized in Table 1. These results demonstrated that a ratio between 1:0.1 and 1:0.05 would give the optimum coverage with no separate MNP in the sample.

**Table 1:** Summary of Product Characteristics for Different Molar Ratios of BNNS:CoFe_2_O_4_

BNNS:CoFe_2_O_4_ molar ratio	coverage of BNNS	magnetization (A m^2^ kg^–1^)
1:0.1	high, some separate MNP	27
1:0.05	medium; no separate MNP	17
1:0.01	low; no separate MNP	6

### Membrane Preparation and Surface Area Analysis

Membranes of BNNS, BNNS–Fe_3_O_4_, and BNNS–CoFe_2_O_4_ were made by filtering an aqueous suspension on a polyvinylidene fluoride (PVDF) 0.45 μm filter on fritted glass filtering apparatus. Thus, nanosheet membranes were formed on the PVDF substrate. SEM images of the edge profile of each of the membranes were taken to show the layering. The SEM images (Figure S22A–C) show the uncoated BN, the Fe_3_O_4_ coated BNNS at 1:0.05 ratio, and the CoFe_2_O_4_ coated BNNS at 1:0.05 ratio, respectively. As can be seen, excellent coverage has been achieved for the BNNS coated with MNPs.

In advance of absorption and filtration studies, BET analysis was carried out on the BNNS (Figure S23), BNNS–Fe_3_O_4_ (Figure S25), and BNNS–CoFe_2_O_4_ (Figure S27) samples in order to understand the surface area and pore size characteristics. Adsorption performance is closely related to the specific surface area and pore size distribution of a material.^31^ Nanocomposites with a BNNS–MNP ratio of 1:0.05 were chosen for this analysis. Surface areas were calculated from the adsorption branch of the isotherm in the linear region (*P*/*P*_0_: 0.1–0.3). The Barrett–Joyner–Halenda (BJH) method was used to calculate pore size and pore volume from the desorption branch of the isotherm.

The surface areas of the samples ranged from ∼33 to 39 m^2^/g with higher surface areas for the coated samples relative to the uncoated one. The surface area is believed to derive primarily from the morphology of the nanosheets, which comprise multiple layers of sheets with slit-like void spaces in between. Flakes are typically 50 to 1000 nm in width, with corresponding nanometer-scale void spaces between the flakes expected. The higher surface area of the BNNS–Fe_3_O_4_ and BNNS–CoFe_2_O_4_-coated samples is believed to be due to the additional surface area contribution of the Fe_3_O_4_ and CoFe_2_O_4_ nanoparticles relative to smooth, uncoated flakes.

Significant hysteresis was observed in the adsorption–desorption isotherms (all type IV) of the uncoated sample and, to a lesser extent, for the coated samples. BJH pore size distributions for each of the samples showed strong volume adsorption in the measurement range ∼2–30 nm. All samples exhibited a prominent peak ≲5 nm. For uncoated BNNS (Figure S24), the associated pore volume in this size range was only 0.007 cc/g or 3.9% of the 0.18 cc/g total. By contrast, for BNNS–Fe_3_O_4_ (Figure S26), the pore volume was 0.013 of 0.12 cc/g, i.e., 11.2%, and for BNNS–CoFe_2_O_4_ (Figure S28), the pore volume was 0.012 of 0.10 cc/g, i.e., 10.3%. The much higher prevalence of sub-5 nm pores for the coated samples is believed to be due to light agglomeration of Fe_3_O_4_ and CoFe_2_O_4_ nanoparticles, resulting in the creation of additional void spaces. On the other hand, for the uncoated sample, the void spaces between the primary BN nanoflakes are the sole contributors to the surface area.

From SEM analysis, a broad size range of void spaces was observed between the BN nanoflakes. For BNNS–Fe_3_O_4_ and BNNS–CoFe_2_O_4_, the flakes are coated with nanoparticles and it appears that a proportion of the interflake voids are partially filled with nanoparticles. A void space will have a certain surface area and pore volume, whereas an equivalent void space filled with loosely agglomerated nanoparticles will have a higher surface area and a lower pore volume. In fact, the total pore volumes of the coated and uncoated samples revealed a significant difference, with the value of 0.18 cc/g for BNNS being considerably larger (≥50%) than that of the coated samples at 0.12 cc/g and 0.10 cc/g for BNNS–Fe_3_O_4_ and BNNS–CoFe_2_O_4_. These lower pore volumes can be attributed to nanoparticles coating the BN flakes and partially filling the slit void spaces between the flakes. The surface area and pore volume results are summarized in Table 2.

**Table 2:** Summary of the BET Surface Area and Pore Volume Data

sample	surface area (m^2^/g)	total pore volume (cc/g)	% porosity (<5 nm)
BNNS	32.9	0.18	3.9
BNNS–Fe_3_O_4_	38.8	0.12	11.2
BNNS–CoFe_2_O_4_	34.6	0.10	10.3

### Testing the Membranes for Filtration Applications

Freshly prepared membranes with a BNNS:MNP ratio of 1:0.05 have been tested for retention of methylene blue (MB) dye to see the ability of the membranes to remove a pollutant dye. A PVDF membrane was used as a support to create the BNNS membrane, and it was used in combination with the BNNS for filtration. As a reference, the PVDF membrane on the fritted glass filtering apparatus was used on its own to filter MB and it showed minimal ability to capture the MB as expected, which will be discussed later. The PVDF membrane had a thickness of 0.105 mm and a mass of 125 mg on average.

The preparation of the membrane for testing is described in the experimental section. This gave a membrane with a size of 0.001018 m^2^ (36 mm diameter). For each of the membranes, the BNNS, BNNS–Fe_3_O_4_, and BNNS–CoFe_2_O_4_ membranes, a mass of 40 mg of the material was used to create the membrane. The thickness of the membrane in all cases was similar with 0.050 mm for BNNS, 0.052 mm for BNNS–Fe_3_O_4_, and 0.053 mm for BNNS–CoFe_2_O_4_ as measured using a micrometer screw gauge.

Flow rates for the prepared membranes were calculated at 1 bar pressure using MP H_2_O (Millipore Water). The PVDF membrane had a flow rate of 26,700 L m^–2^ h^–1^, which is higher than a specified value from a manufacturer of these membranes.^32^ The BNNS membrane in combination with the PVDF had a flow rate of 620 L m^–2^ h^–1^. The BNNS–Fe_3_O_4_ membrane in combination with the PVDF had a flow rate of 343 L m^–2^ h^–1^. The BNNS–CoFe_2_O_4_ membrane in combination with the PVDF had a flow rate of 492 L m^–2^ h^–1^. The reduction in flow rates for the Fe_3_O_4_ and CoFe_2_O_4_-coated BNNS is indicative of smaller pores in the membrane, as the particles on the surface of the BNNS would create smaller pores for the liquid to filter through, thereby decreasing the flow rates. These membrane characteristics are summarized in Table S1.

The prepared membranes were tested for retention of methylene blue (MB) dye. The % removal for each aliquot can be calculated using the Beer–Lambert law. This was done for the BNNS, the BNNS–Fe_3_O_4_ and the BNNS–CoFe_3_O_4_ (Table S2). It was found that prior to becoming saturated, the membrane retained >99% of the MB dye from solution. This is excellent performance. In addition, the magnetic modality gives the possibility for in situ magnetic inductive heating of the membrane that could be used for membrane regeneration once it has become saturated or blocked. The saturation point is easily observed for MB using UV–vis spectroscopy analysis. For the testing of the membranes, a 40 mg membrane was made on the PVDF support. Then, 20 mL aliquots of a 21.9 μM solution of MB were filtered through the prepared membrane using vacuum filtration, giving an effective membrane pressure of 1 bar approximately. For the BNNS membrane, 20 mL took 114 s, for the BNNS–Fe_3_O_4_ membrane, it took 206 s, and for the BNNS–CoFe_2_O_4_ membrane, it took 143 s. The initial deep blue solution was observed to become clear as filtrates were collected, as can be seen in the image for the BNNS membrane (Figure S29). As can be seen in the image, after the fifth aliquot, the filtrate begins to show the blue of the MB as the membrane becomes saturated.

**Figure 6:**
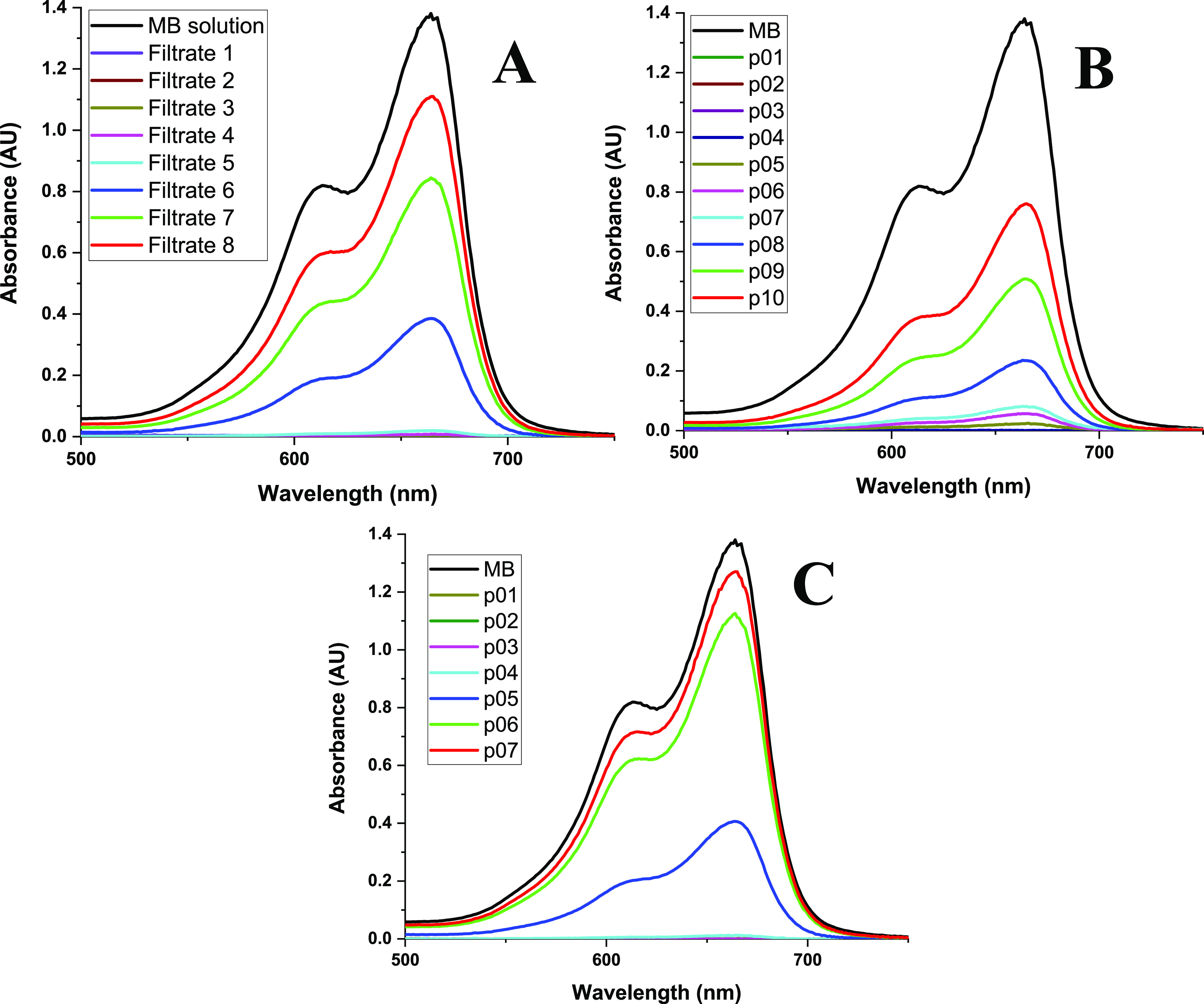
UV–vis analysis of successive 20 mL aliquots of MB solution for (A) BNNS membrane, (B) BNNS–Fe_3_O_4_ membrane, and (C) BNNS–CoFe_2_O_4_. See also Table S2 for numerical values.

These results were validated with UV–vis spectral analysis for the BNNS membrane as can be seen in Figure 6A. It can be seen that for the first 4 runs, there is >99% removal of the dye and then at the fifth run, the MB begins to pass through the membrane as the membrane is getting saturated. By the eighth filtrate, the solution is approaching the concentration of the original MB solution before filtration as the membrane is now becoming saturated and the adsorption capacity reaches its limit.

The same procedure was applied to the BNNS–Fe_3_O_4_ membrane with successive 20 mL aliquots of the 21.9 μM MB solution. These filtered aliquots were collected (Figure S30). It can clearly be seen how the initial clear filtrate begins to have MB in it with successive filtrations through the same membrane. These results again were quantified with UV–vis spectroscopy analysis, as can be seen in Figure 6B.

The BNNS–Fe_3_O_4_ membrane (40 mg) and 20 mL aliquots of a 21.9 μM MB solution of MB with a molar mass of 373.9 g mol^–1^ were used. Each 20 mL will contain 0.157 mg of MB (0.02 L × 0.021.9 mM × 373.9 g mol^–1^) that will be absorbed until the membrane is saturated. The graph (Figure S31) shows this and the beginning of the plateau of the curve, which will plateau at approximately 1.5 mg. This gives a capture of 1.5 mg for 40 mg of membrane or 37.5 mg g^–1^, which is lower than other previously reported values for adsorption of MB with pure BN^4^.

The same procedure was used for the BNNS–CoFe_2_O_4_ membrane with successive 20 mL aliquots of the 21.9 μM MB solution. These results again were quantified with UV–vis spectroscopy analysis, as can be seen in Figure 6C. The same type of calculation for saturation for this material gave a value of up to 23.6 mg g^–1^. A similar calculation for the BNNS membrane show that the 40 mg of BNNS adsorbs 1.0 mg when plateauing, giving 25.0 mg g^–1^, which again is lower than other reported values for pure BN.^4^

According to the UV–vis analysis, the BNNS membrane captures more MB from solution initially as the fifth filtrate for this has the lowest absorbance at 0.019 AU in comparison with the BNNS–Fe_3_O_4_ and BNNS–CoFe_2_O_4_ for their fifth filtrate, 0.025 AU and 0.406 AU, respectively. Then, the BNNS membrane saturates quickly while the BNNS–Fe_3_O_4_ saturates slowly and so adsorbs more MB overall. Apart from this, all membranes showed a tendency toward saturation and then letting the MB through the filter. With the MNPs as part of the membrane, the potential for magnetic heating and regeneration as the MB is captured by the membrane is possible.

As mentioned previously, the PVDF membrane was tested for retention of the MB dye on its own. A similar calculation using 20 mL aliquots of the 21.9 μM MB solution showed that the PVDF membrane alone adsorbs 0.1 mg when plateauing, equating to 2.5 mg g^–1^. This value then has to be subtracted from the value from each of the membranes to give the final values for the BNNS and the BNNS–MNP composites. These results are summarized in Table 3.

**Table 3:** Adsorption Capacities for Various BN Morphologies from Pervious Publications and Our Work

BN material	adsorption (mg g^–1^)	reference
BN spheres	233	(33)
BN nanocarpets	272	(34)
porous BN	313	(35)
BN hollow spheres	117	(36)
BNNS	22.5	this work
BNNS–Fe_3_O_4_	35.0	this work
BNNS–CoFe_2_O_4_	21.1	this work

BN of different morphologies has been tested previously for removal of MB from aqueous solution. Different morphologies showed different adsorption capacities, with some examples of the adsorption capacities for removal of MB from aqueous solution given in Table 3.

### Testing the BNNS–CoFe_2_O_4_ for Regeneration and Recycling

For water remediation applications, recyclability of the adsorbent is an important factor for sustainability and environmental safety.^14^ The recyclability of the BNNS–CoFe_2_O_4_ nanocomposites was tested in order to determine whether it could be a potential reusable filtration membrane. The process involved passing the MB solution through the membrane in successive 20 mL aliquots until membrane saturation was reached. Then, the membrane was washed with methanol and water. The membrane was then sonicated in acetone to remove the nanocomposite from the PVDF support. The material was magnetically extracted from the acetone and placed in a furnace at 400 °C to burn off any remaining MB and organics. Then, the nanocomposite was sonicated in water to make a membrane again from the same material and reused to filter the MB solution again. The nanocomposite was recycled 7 times, and the percent MB removed was calculated from the UV–vis analysis using the Beer–Lambert law for each recycle. A plot of the mg/g for MB removal against the recycle number is shown (Figure S32). As can be seen, there is variability between the runs for the amount of MB adsorbed with the third run capturing the highest at 23.6 mg/g. As the same material was recycled, with removal from the membrane and magnetic extraction, some material was lost between runs. The mass of the sample was measured after being in the furnace each time and it showed a small loss of material after each run, which is shown (Table S3). Even though there was a loss of material on each run, the adsorption increased up to the second recycle. After that, there was a general trend in reduction of adsorption with variability between runs. This indicates that other factors may be involved, such as how the material packed on the PVDF support while the membrane is being formed. This material shows MB removal after several recycles, indicating that this nanocomposite has the potential for practical applications in water purification to remove MB from wastewater. Future studies will be done to develop magnetic induction heating that can be used to remove adsorbed dyes and organic impurities.

### Filtration and Removal of Magnetic Nanoparticles

One final test that was done with the BNNS membrane was to see if it could filter MNPs. Magnetite MNPs of 14 ± 5 nm were prepared by a simple coprecipitation method as described in the experimental section. A TEM image (Figure S33) confirmed the size of the particles. Bare MNPs are prone to agglomeration due the lack of a surfactant because of the tendency of the particle to reduce the surface energy. These MNPs were tested for filtration with the BNNS membrane. The procedure involved sonication of a 0.2 mg/mL solution of the MNPs to create well-dispersed solutions. Then, 20 mL of this solution was filtered at 1 bar pressure. The filtrate was then left on a magnet overnight to see if any particles came out of the solution. For the BNNS membrane, there were no magnetic particles extracted on the magnet, indicating that no particles passed the membrane. The membranes had captured all (100%) MNPs. The surface of the membranes could be seen to have the MNPs on it (Figure S34).

## Conclusions

Thus, we have shown that BNNS can be successfully coated with MNPs to give excellent coverage that is robust and stable to sonication. This was achieved using the exfoliated 2D BNNS, which were transferred to ethylene glycol and reacted with the metal precursors. TEM and SEM imaging has proven the abundant presence of MNPs of Fe_3_O_4_ and CoFe_2_O_4_ on the surface of BNNS. FTIR and XPS have indicated the formation of bonds between the MNPs and the BNNS. The magnetic properties of the nanocomposites were reasonable with the nanosheets being capable of extraction from solution with a neodymium magnet. The coverage achieved for the BNNS–Fe_3_O_4_ material as observed by TEM and SEM was higher than any previously reported case in the literature. Changing the ratio of the MNP to the BNNS resulted in more or less coverages depending on the ratio. The synthesized nanocomposites have been used to produce membranes for nanofiltration applications.

The membranes were tested for filtration of MB, and it was found that the membranes absorb the MB until it saturates, at which point the MB is found in the filtrate. The capture of the MB blue of >99% prior to saturation beginning is a promising result. Our future work will involve the testing of the membranes using magnetic induction heating to see if the dyes or other organic pollutants can be burned out to recycle the filter. This should open unique approaches for the production of efficient membranes incorporating magnetic functionality. The addition of the magnetic functionality should also enable the membranes to be potentially regenerated using magnetic heating.

## Supporting Information Available

TEM & SEM images of exfoliated BNNS and BNNS–Fe_3_O_4_; size distribution histograms; FTIR spectra of BN powder, exfoliated BNNS, CoFe_2_O_4_ MNPs, BNNS–Fe_3_O_4_, Fe_3_O_4_ MNPs, and BNNS–CoFe_2_O_4_ nanocomposites; EDX maps of BNNS–Fe_3_O_4_ and BNNS–CoFe_2_O_4_; XPS survey spectra of BNNS, BNNS–Fe_3_O_4_, and BNNS–CoFe_2_O_4_; XPS high-resolution spectra of B1s for BNNS, BNNS–Fe_3_O_4_, and BNNS–CoFe_2_O_4_; phase quantification of BNNS–Fe_3_O_4_ and BNNS–CoFe_2_O_4_; photos of collected filtrates for BNNS membrane and BNNS-Fe_3_O_4_ membrane; VSM graphs for BNNS-Fe_3_O_4_ and BNNS–CoFe_2_O_4_ (PDF)
