# Supplementary material for: Boron Nitride Nanosheets Functionalized with Fe3O4 and CoFe2O4 Magnetic Nanoparticles for Nanofiltration Applications
Source: ACS Appl Nano Mater. 2023 Jun 22;6(13):12526–36. doi: 10.1021/acsanm.3c02375 (PMC10352960; doi:10.1021/acsanm.3c02375)
Supplement: Supplementary file 1 — an3c02375_si_001.pdf [file an3c02375_si_001.pdf]

Supporting Information

Boron Nitride Nanosheets Functionalized with Fe_3O_4 and $CoFe_2O_4$ Magnetic Nanoparticles for Nanofiltration Applications

*Garret Dee[a], Olivia O'Donoghue[a], Aran Rafferty[a], Lee Gannon[b], Cormac McGuinness[b] and Yurii K. Gun'ko[a]**

[a] *School of Chemistry, University of Dublin, Trinity College, Dublin 2, Ireland*

[b] *School of Physics University of Dublin, Trinity College, Dublin 2, Ireland*

Corresponding Author

*Yurii K. Gun'ko - E-Mail: igounko@tcd.ie; ORCID 0000-0002-4772-778X

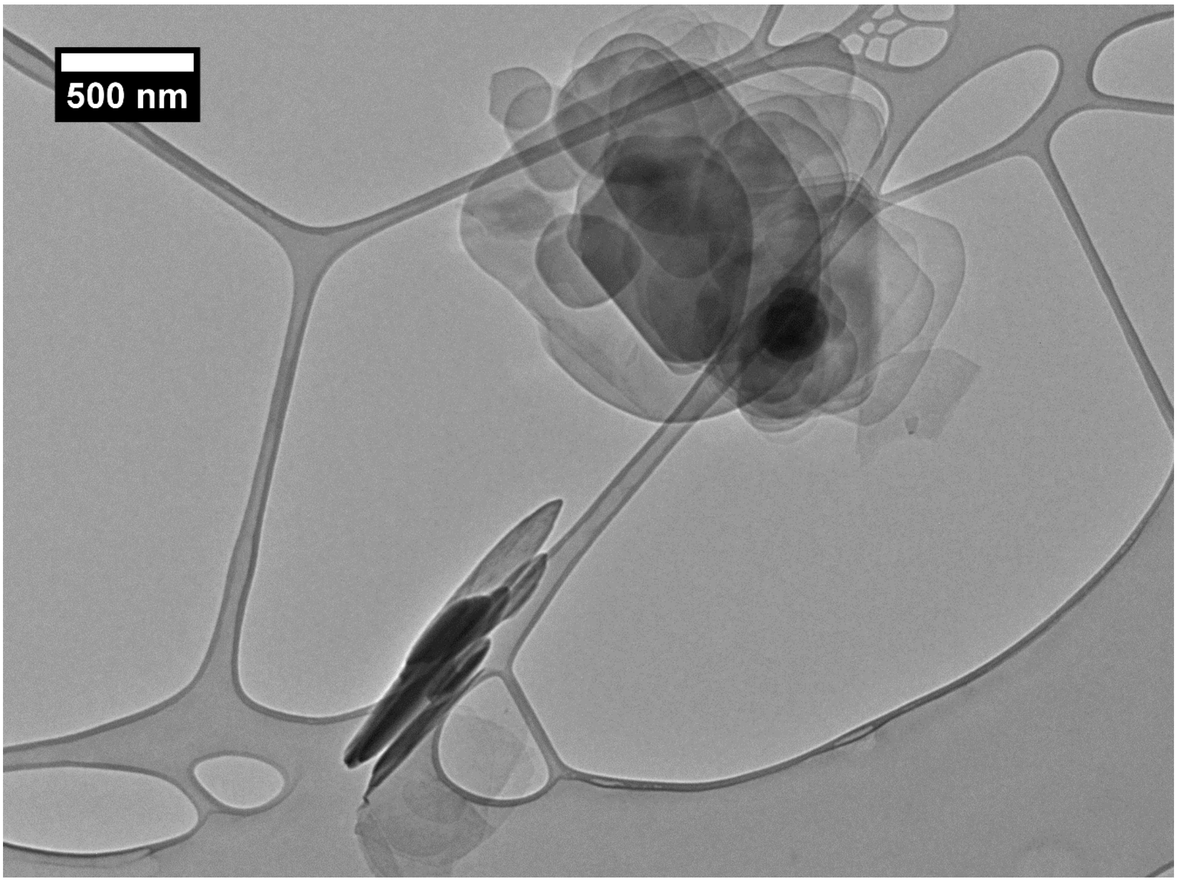

Figure S1: TEM image of exfoliated h-BN to form BNNS showing stacking of individual nanosheets.

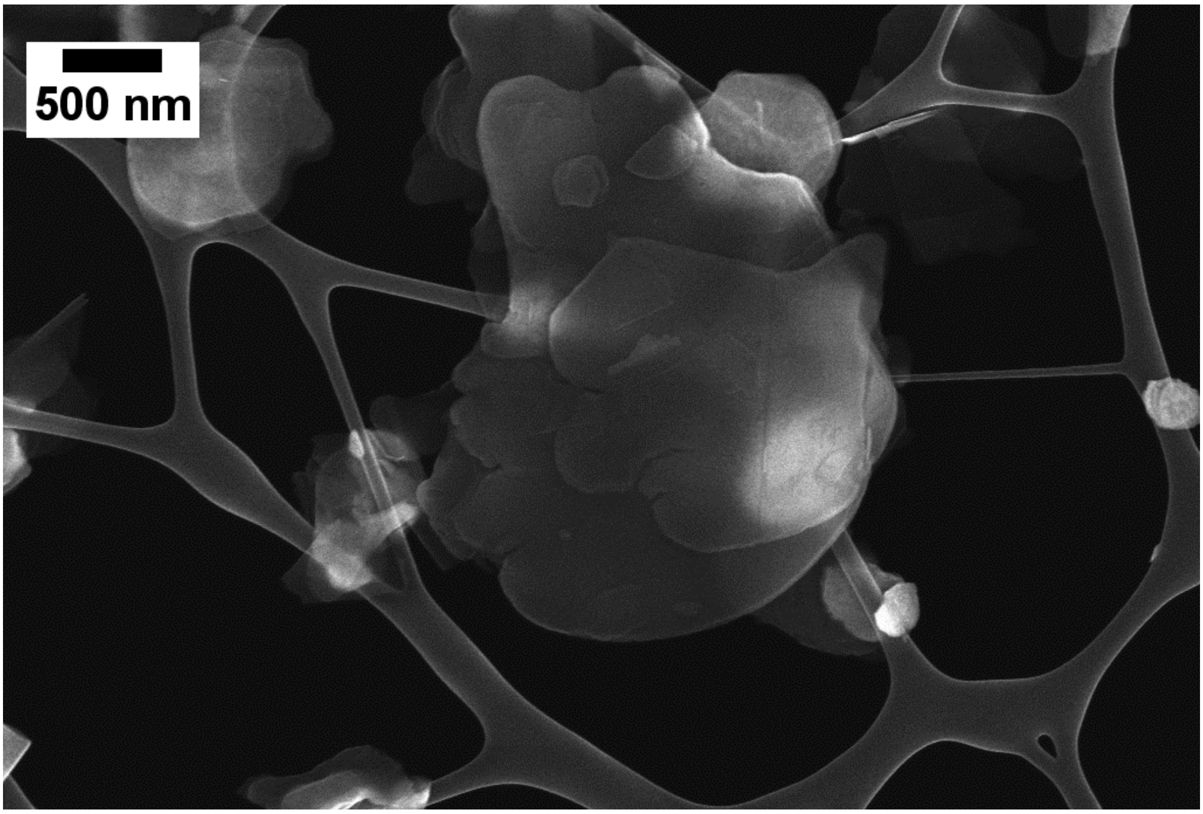

Figure S2: SEM image of exfoliated h-BN to form BNNS showing stacking of individual nanosheets.

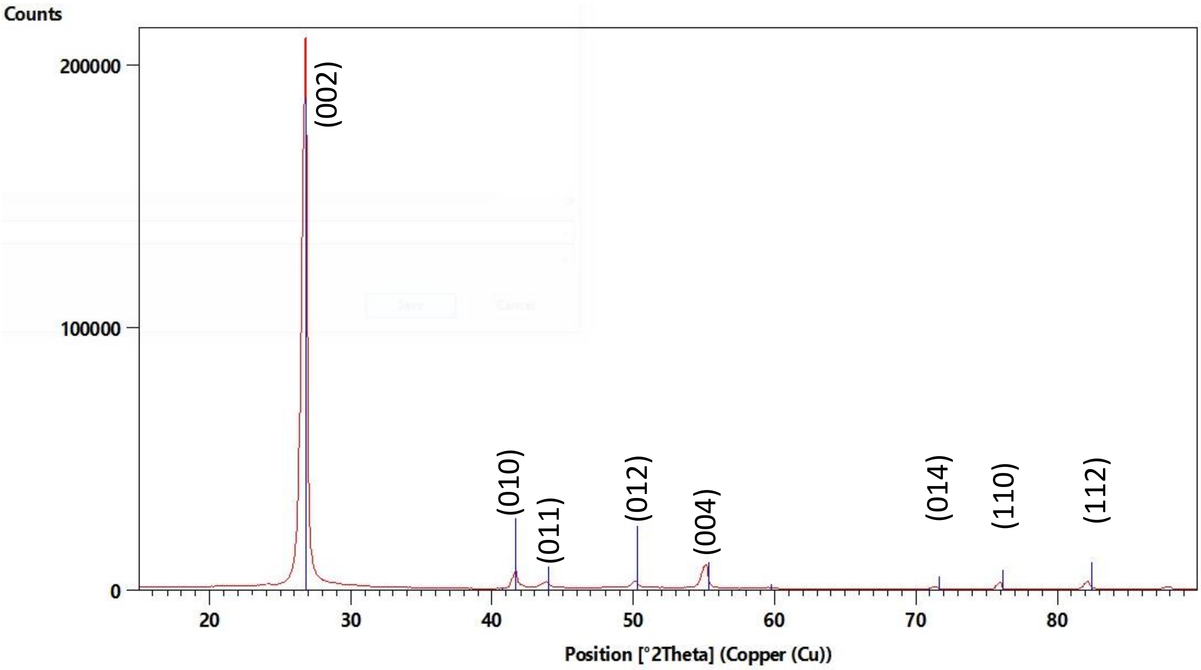

Figure S3: XRD pattern of exfoliated BNNS, showing the hkl planes for the peaks.

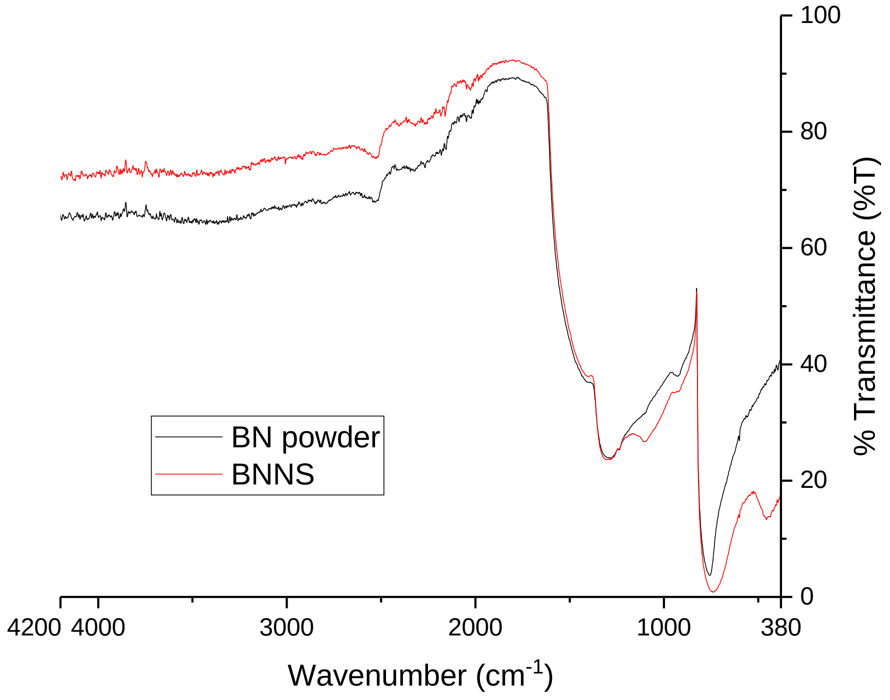

Figure S4: FTIR of BN powder and exfoliated BNNS.

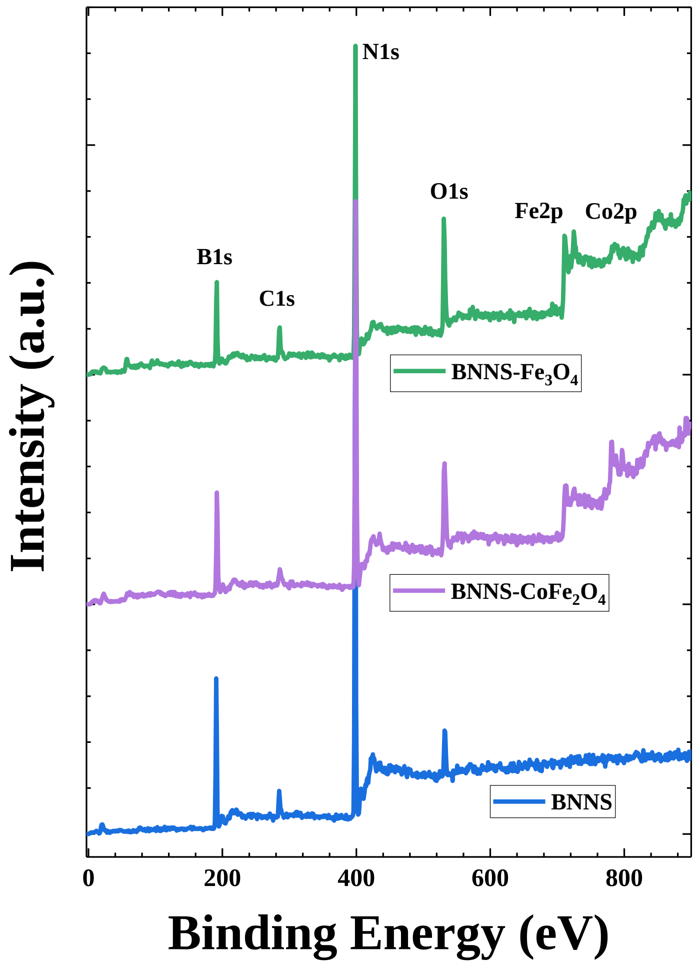

Figure S5: XPS survey spectra of the BNNS, $CoFe_2O_4$, Fe_3O_4, BNNS-$CoFe_2O_4$ and BNNS-Fe_3O_4.

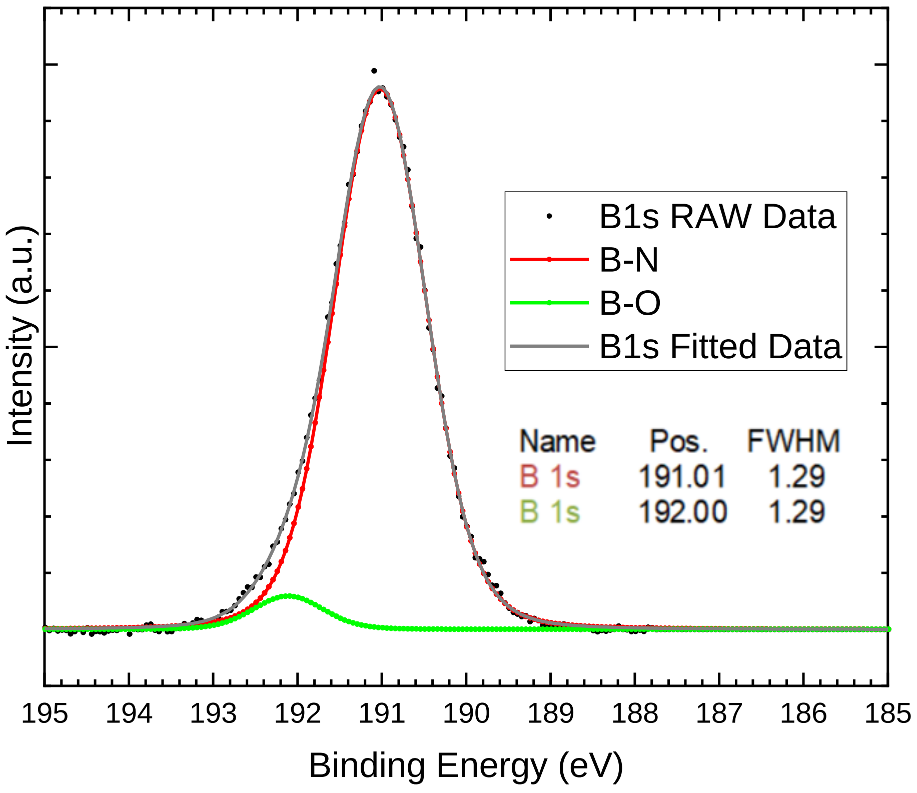

Figure S6: XPS of the BNNS B1s peak showing deconvoluted peaks.

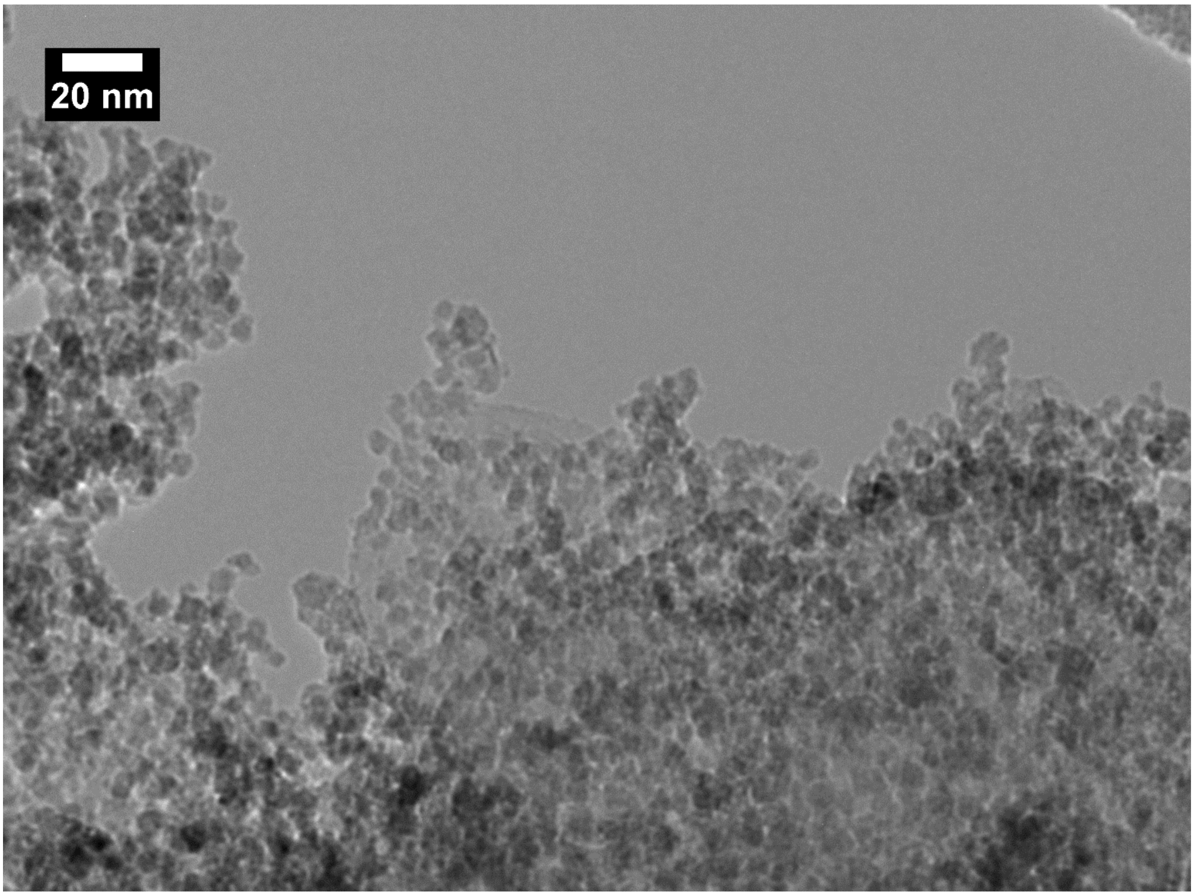

Figure S7: Zoomed in TEM image of Fe_3O_4 particles on the BNNS showing particle sizes

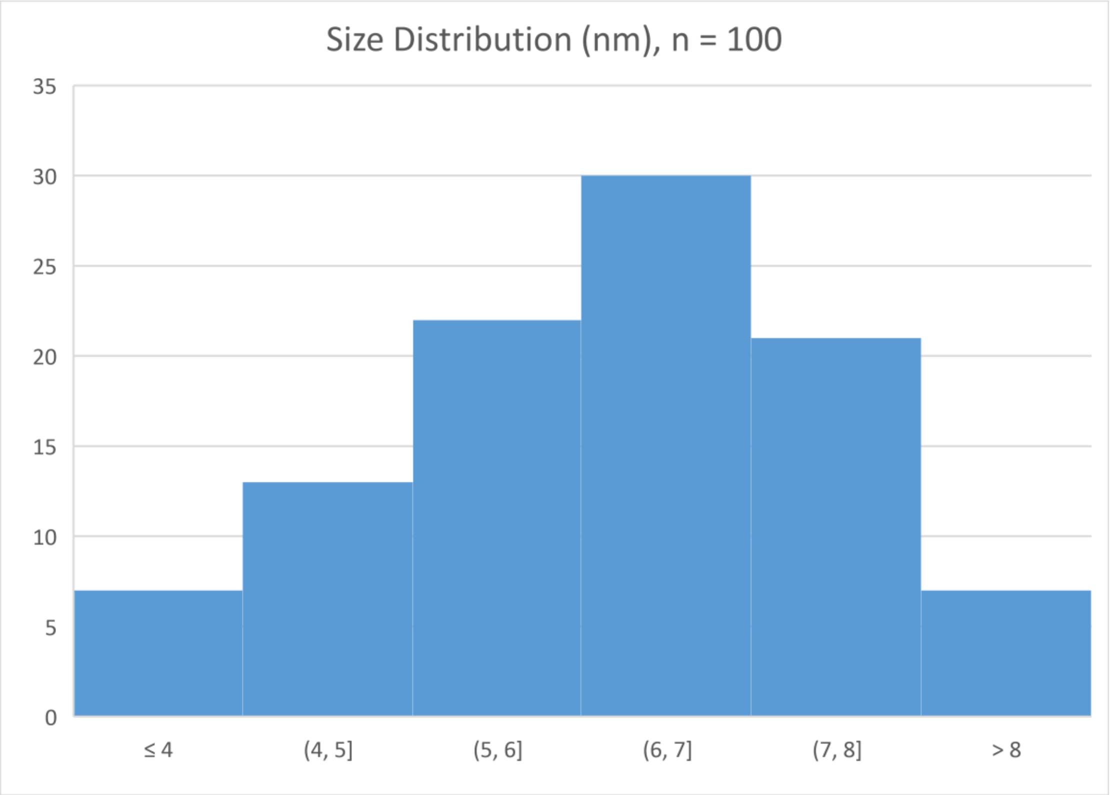

Figure S8: Size distribution for the Fe_3O_4 nanoparticles on the surface of the BNNS.

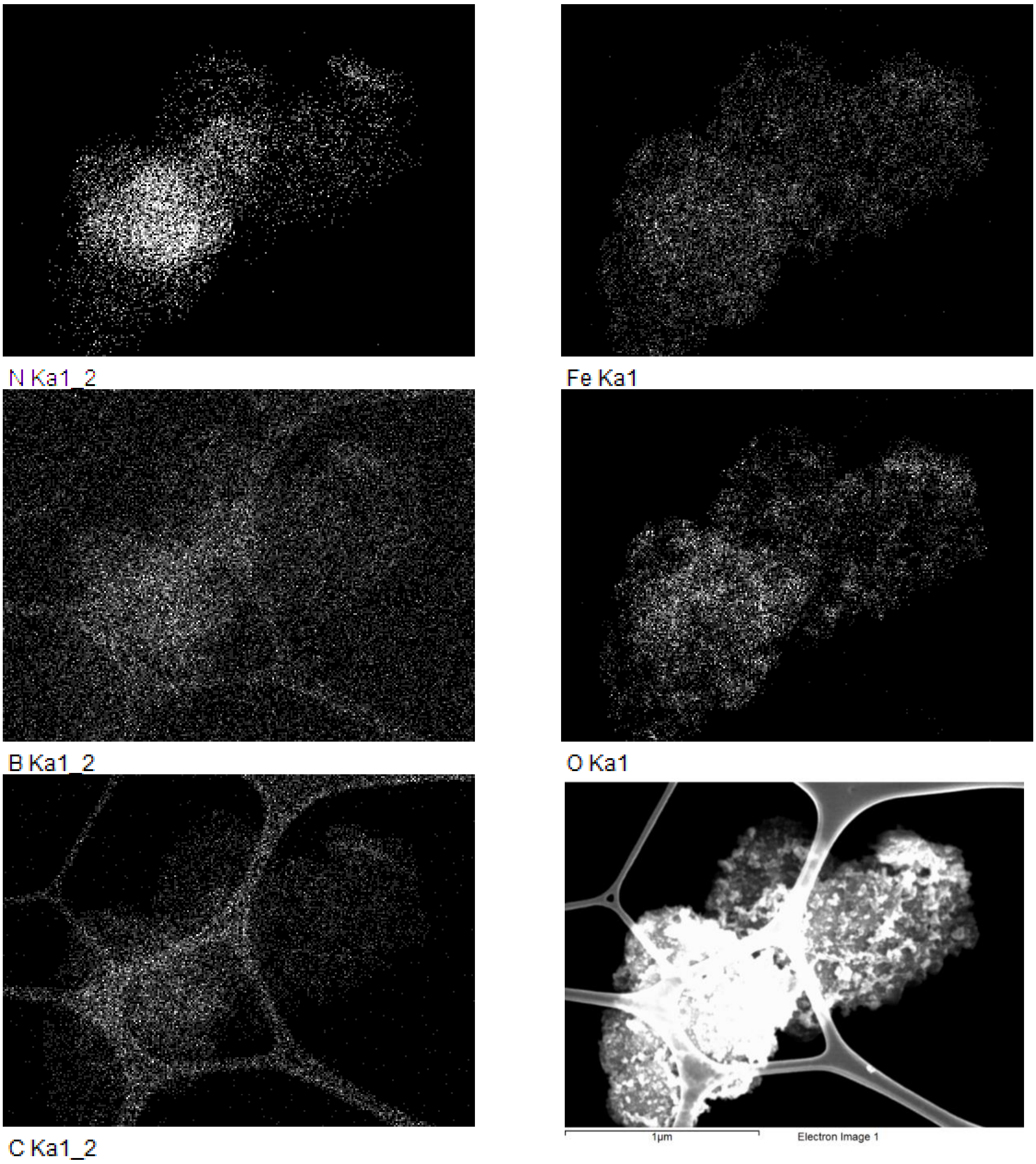

Figure S9: EDX maps of N, B, Fe, O and C showing the detected X-rays from the sample, with the SEM electron image of the sample. Boron is near the limit of the detector and so a lot of background is in this image but the sample can be made out. Carbon shows the Lacey carbon grid clearly and some on the sample, which are possibly traces of alcohol from the washing steps. From the Fe and N analysis, we can clearly see that the Fe_3O_4 coats the BN sheet

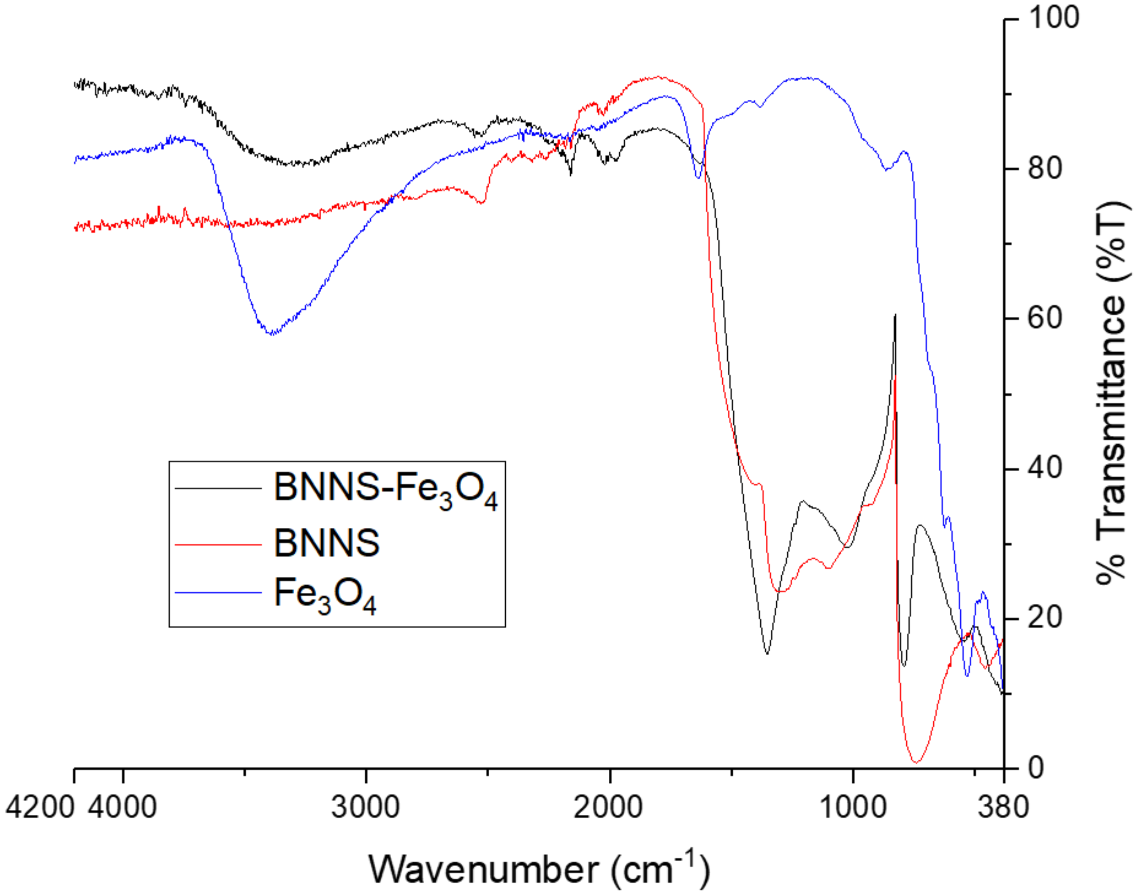

Figure S10: FTIR spectra of BNNS-Fe3O4 with BNNS and Fe3O4 for comparison.

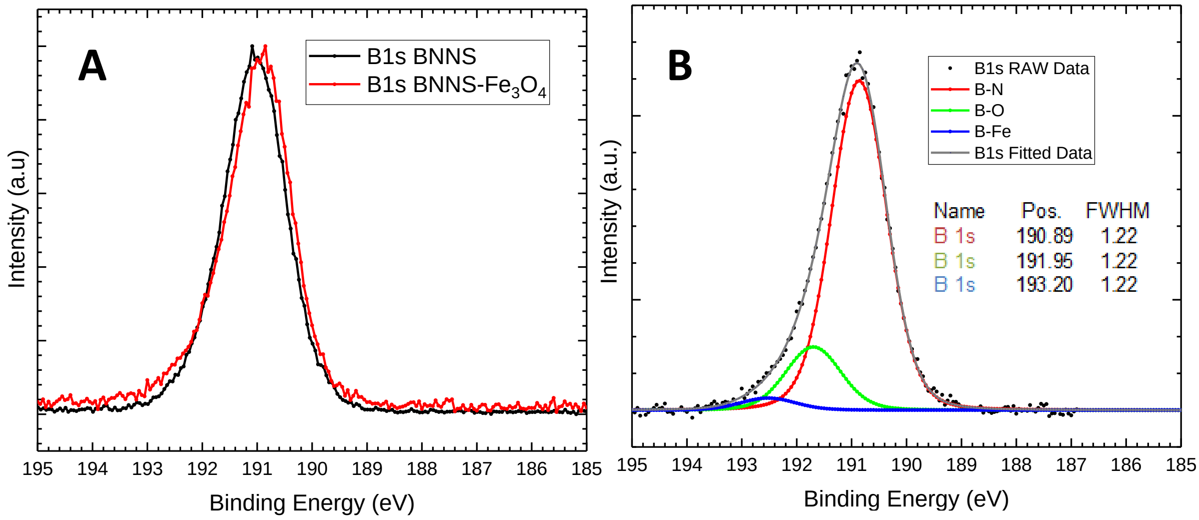

Figure S11: High-resolution XPS spectra (A) B1s of BNNS and BNNS-Fe_3O_4 composite, (B) B1s of BNNS-Fe_3O_4 composite showing fitted and deconvoluted peaks. Note the improvement of the FWHM from Figure S6, indicating that there is less chemical disorder of the B sites in this composite.

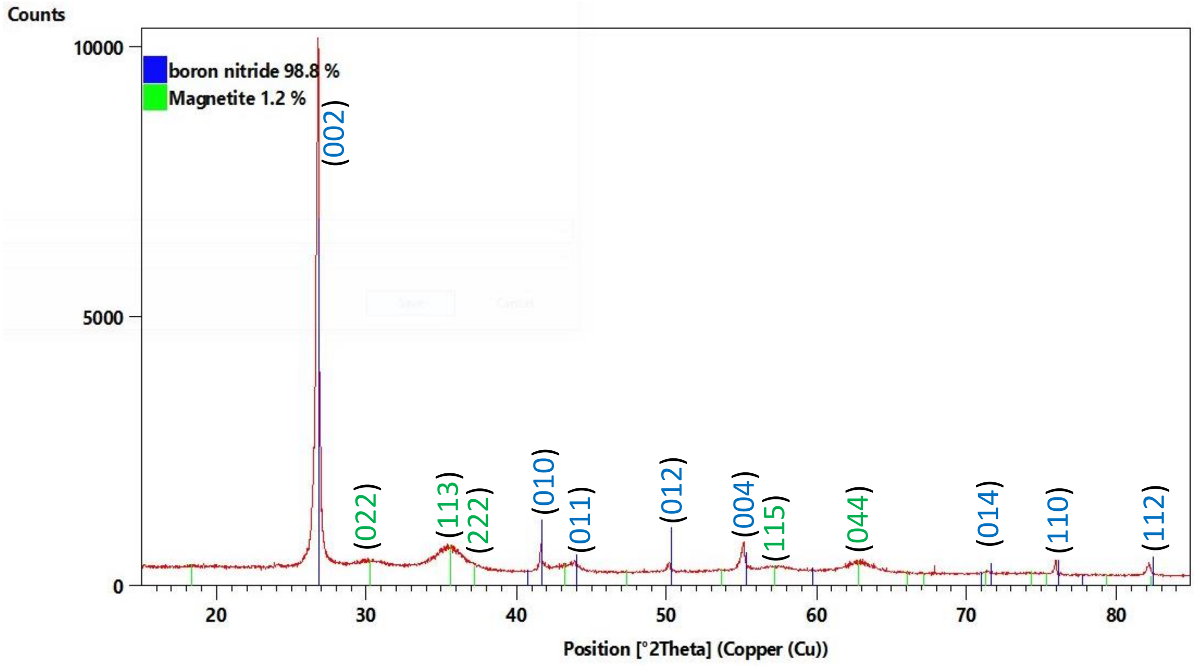

Figure S12: Phase quantification of the BNNS-Fe_3O_4 nanocomposite using HighscorePlus software, showing the hkl planes

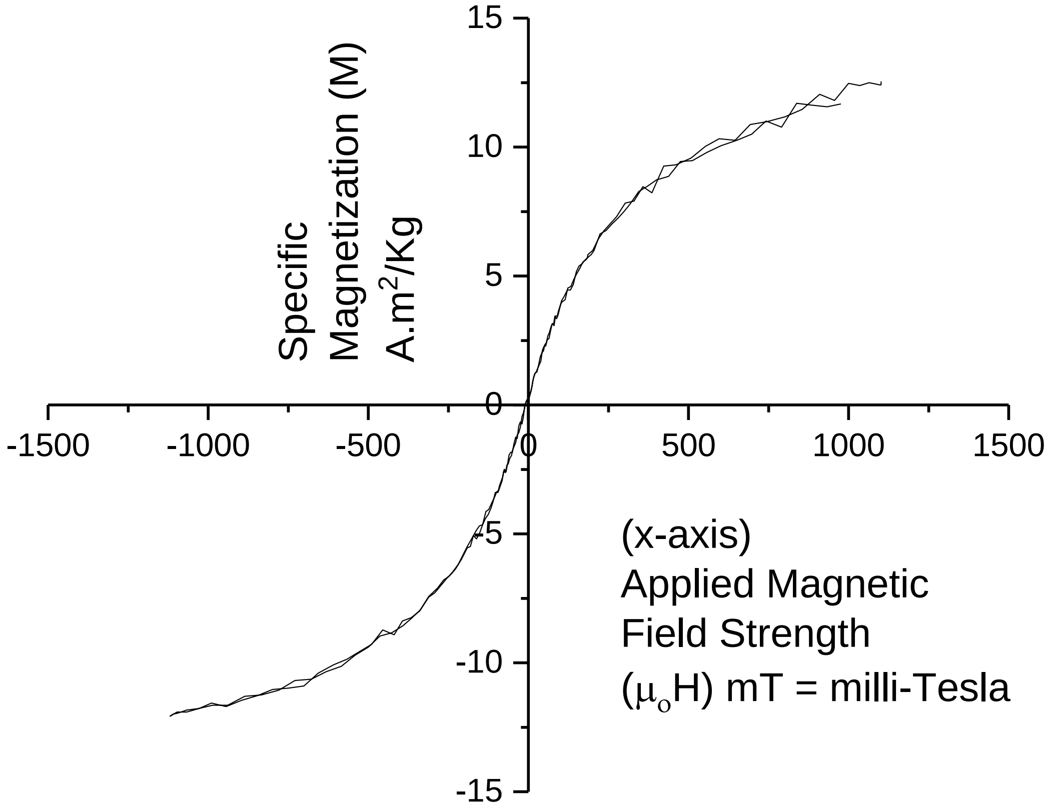

Figure S13: VSM curve of the BNNS-Fe_3O_4 nanocomposites.

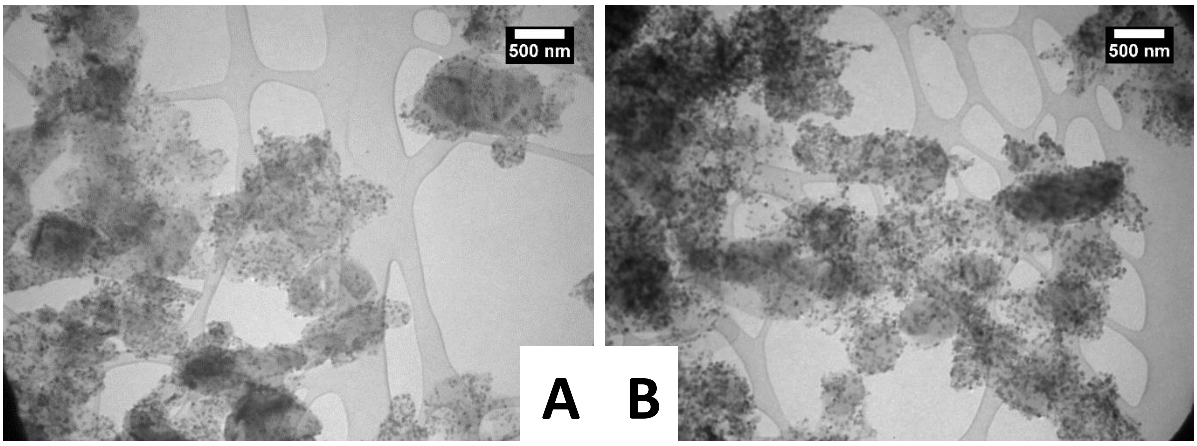

Figure S14: TEM images of Fe_3O_4 coated BNNS when (A) using NaOH and (B) without EDA.

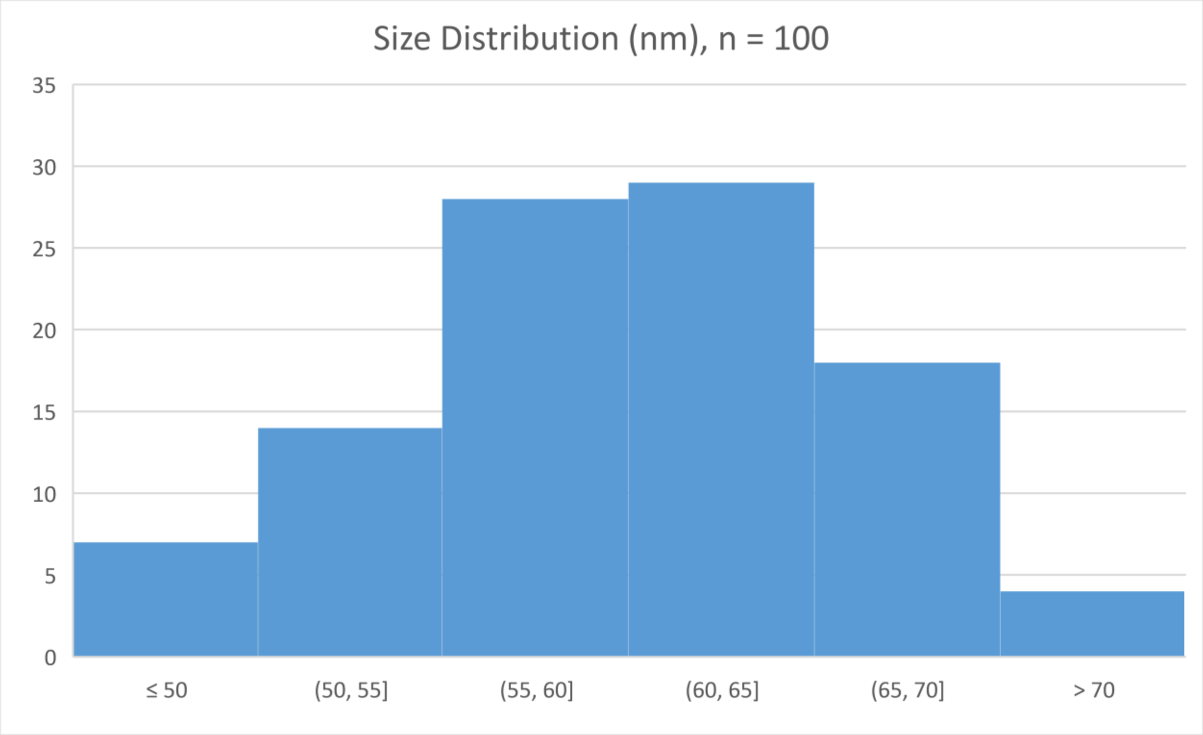

Figure S15: Size distribution for the $CoFe_2O_4$ nanoparticles on the surface of the BNNS.

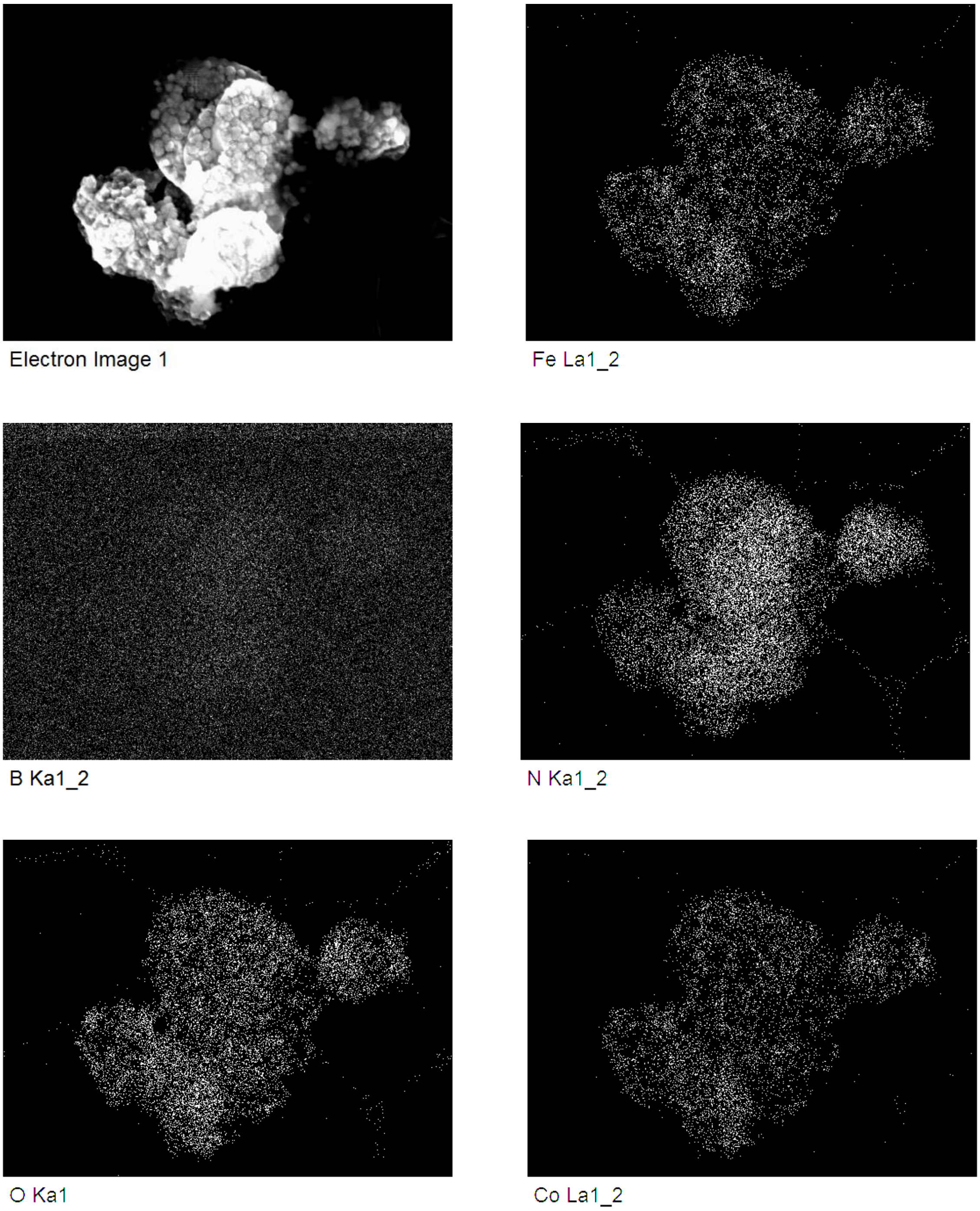

Figure S16: EDX maps of N, B, Fe, O and Co. Again, boron is at the limit of the detector so it show a lot of background. From the Fe, Co and N analysis, we can clearly see that the $CoFe_2O_4$ coats the BN sheet

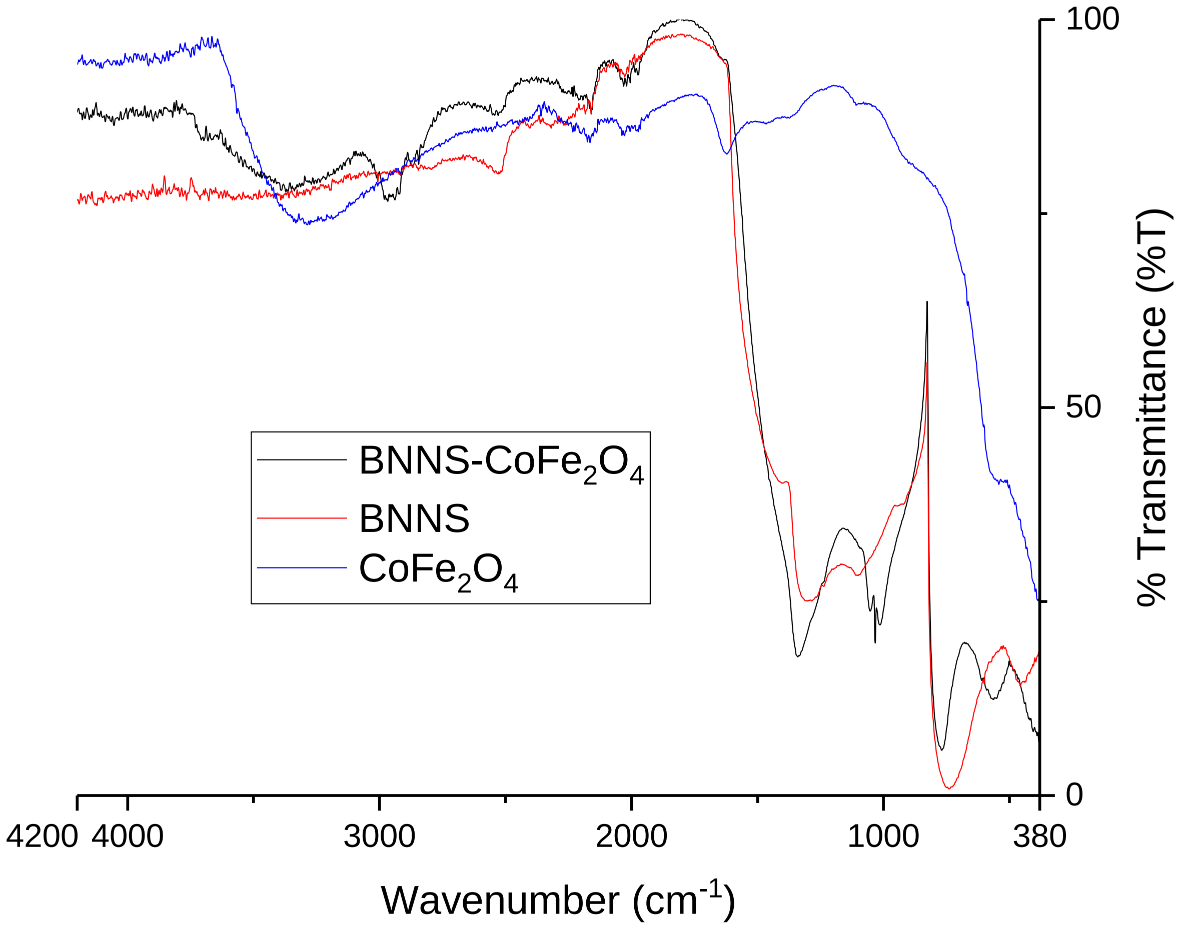

Figure S17: FTIR spectra of BNNS-$CoFe_2O_4$ with BNNS and $CoFe_2O_4$ for comparison.

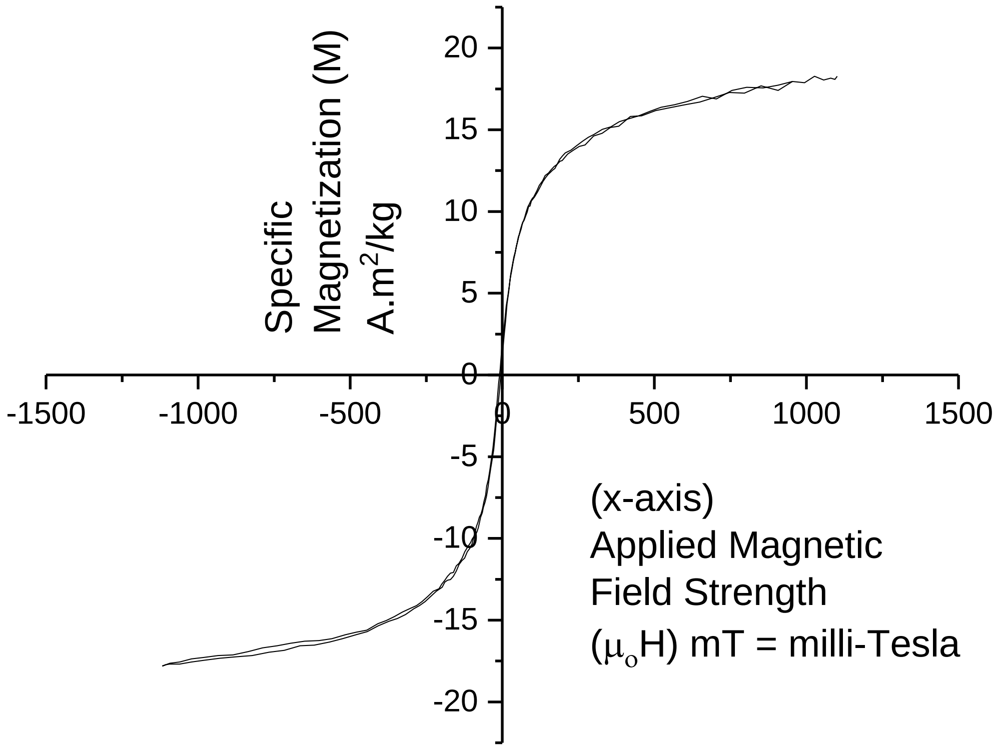

Figure S18: VSM curve of the BNNS-$CoFe_2O_4$ nanocomposites.

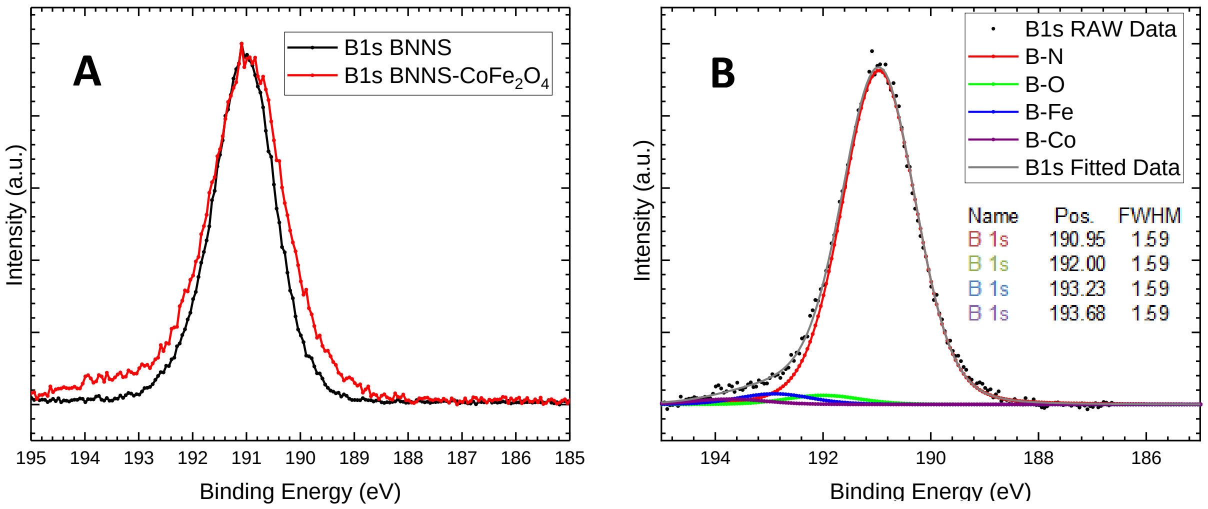

Figure S19: High-resolution XPS spectra (A) B1s of BNNS and BNNS-$CoFe_2O_4$ composite, (B) B1s of BNNS (bottom) and BNNS-$CoFe_2O_4$ composite (top). Note the larger FWHM value from Figure S6, indicating that there is additional chemical disorder of the B sites in this composite.

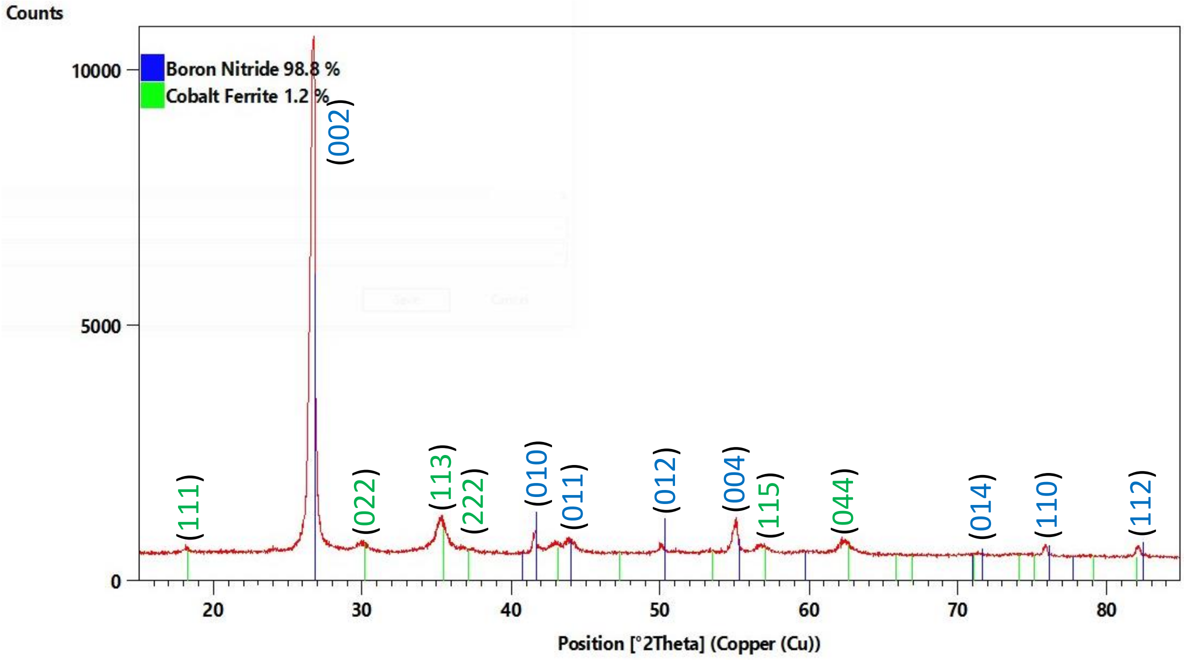

Figure S20: Phase quantification of the BNNS-$CoFe_2O_4$ nanocomposite using HighscorePlus software, showing the hkl planes.

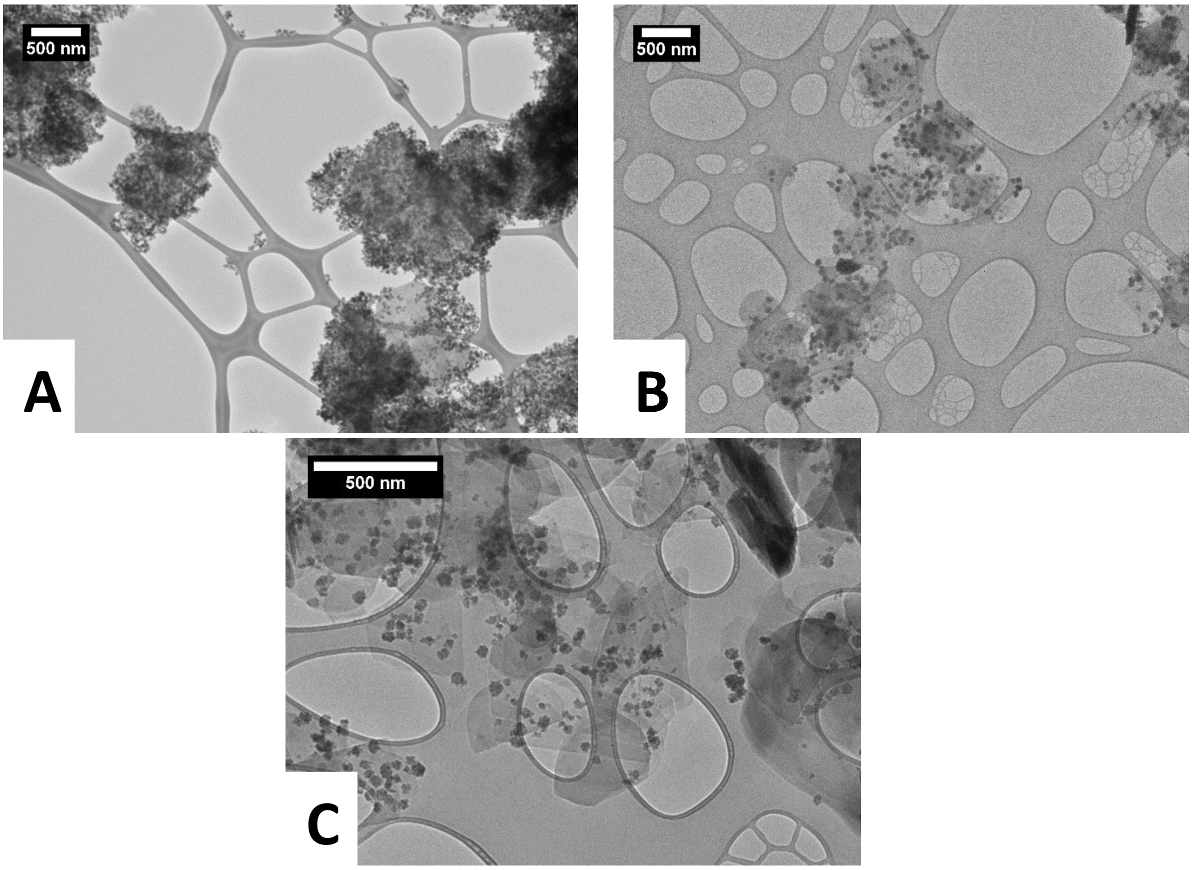

Figure S21: TEM images of the BNNS-$CoFe_2O_4$ with molar ratios of (A) 0.1 (B) 0.05 (C) 0.01

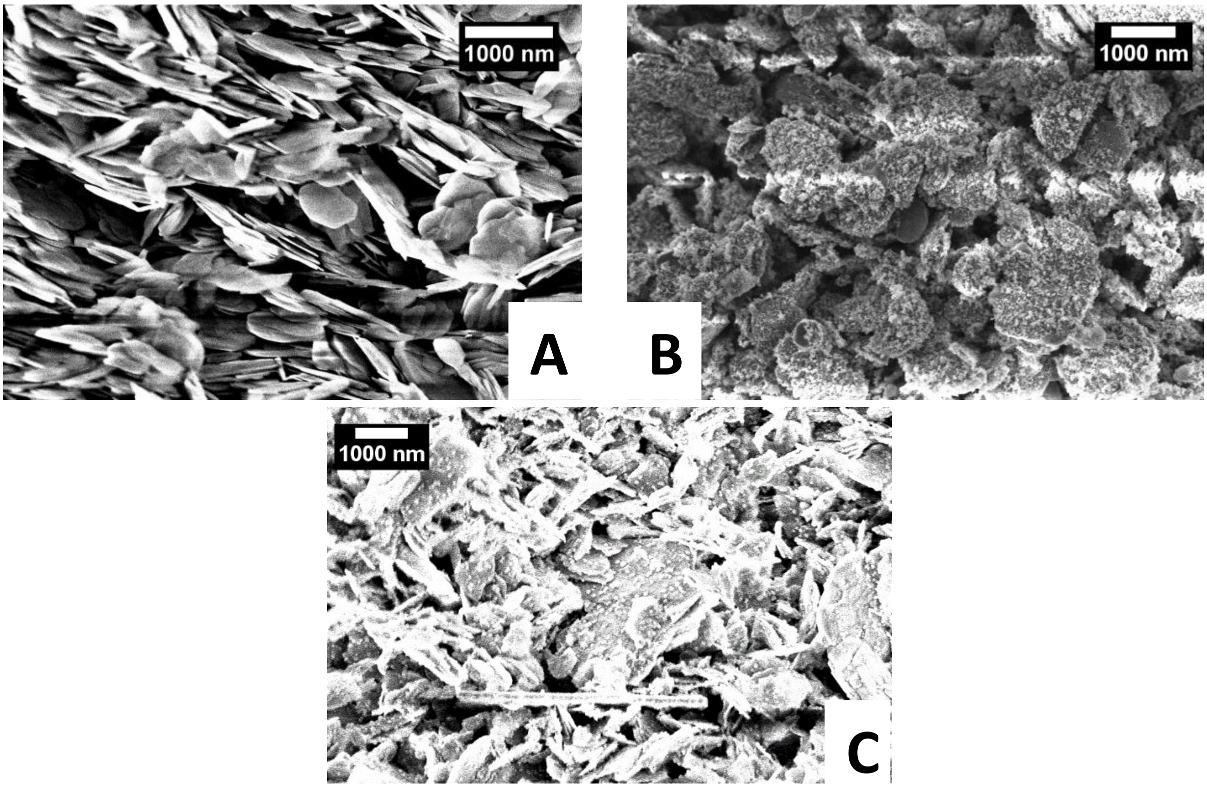

Figure S22: SEM cross-sections images of (A) Bare BNNS membrane (B) Fe_3O_4 coated BNNS membrane and (C) $CoFe_2O_4$ coated BNNS membrane.

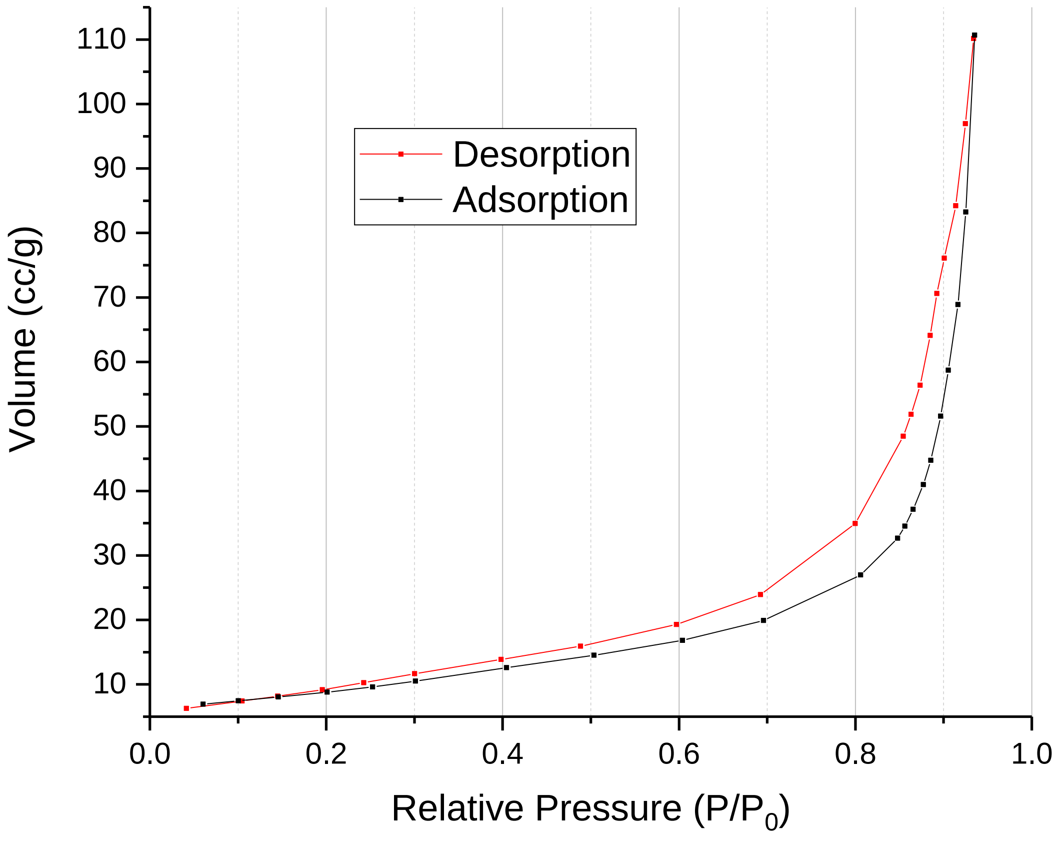

Figure S23: Nitrogen adsorption-desorption isotherm for the BNNS sample.

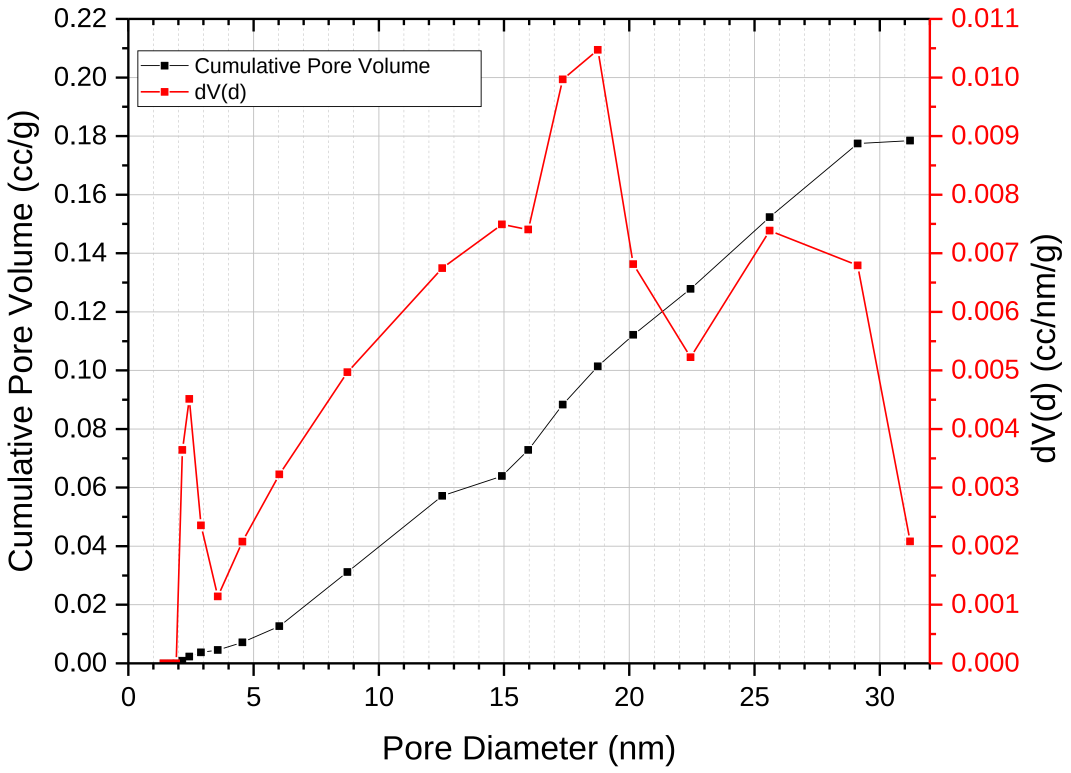

Figure S24: BJH pore size distribution of the BNNS sample.

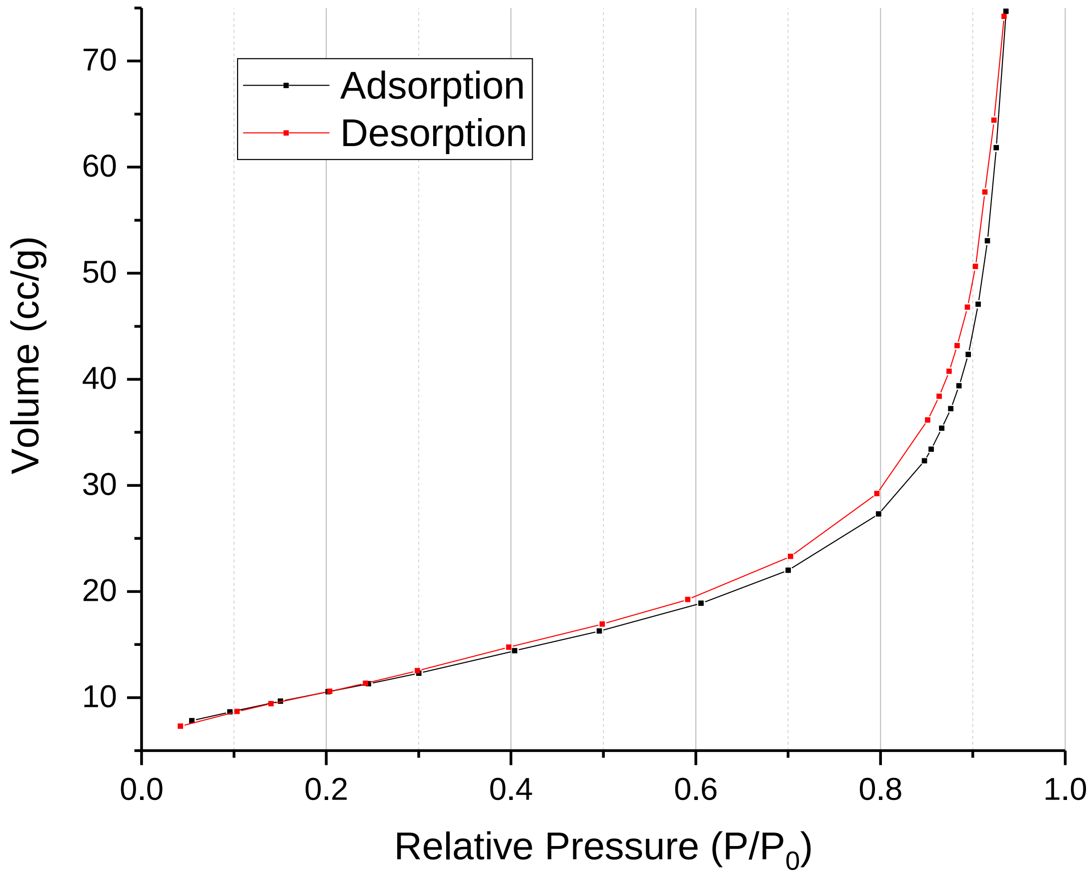

Figure S25: Nitrogen adsorption-desorption isotherm for the BNNS-Fe_3O_4 nanocomposite.

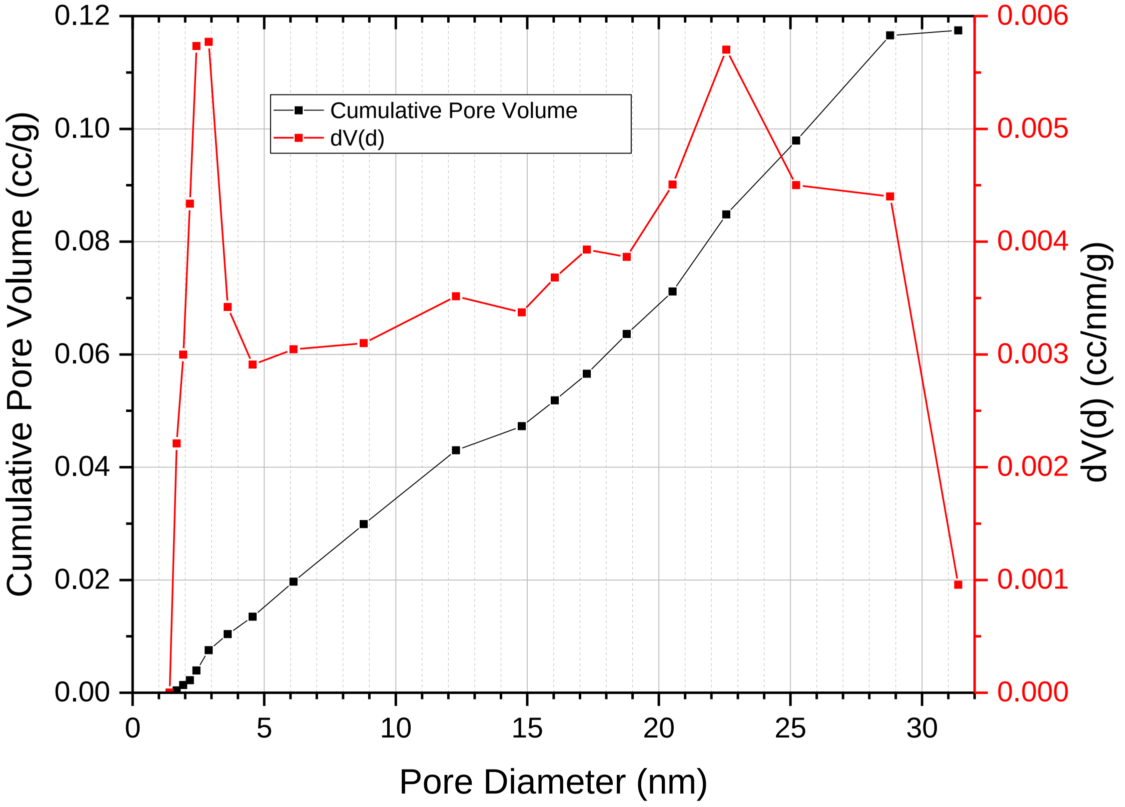

Figure S26: BJH pore size distribution of the BNNS-Fe_3O_4 nanocomposite.

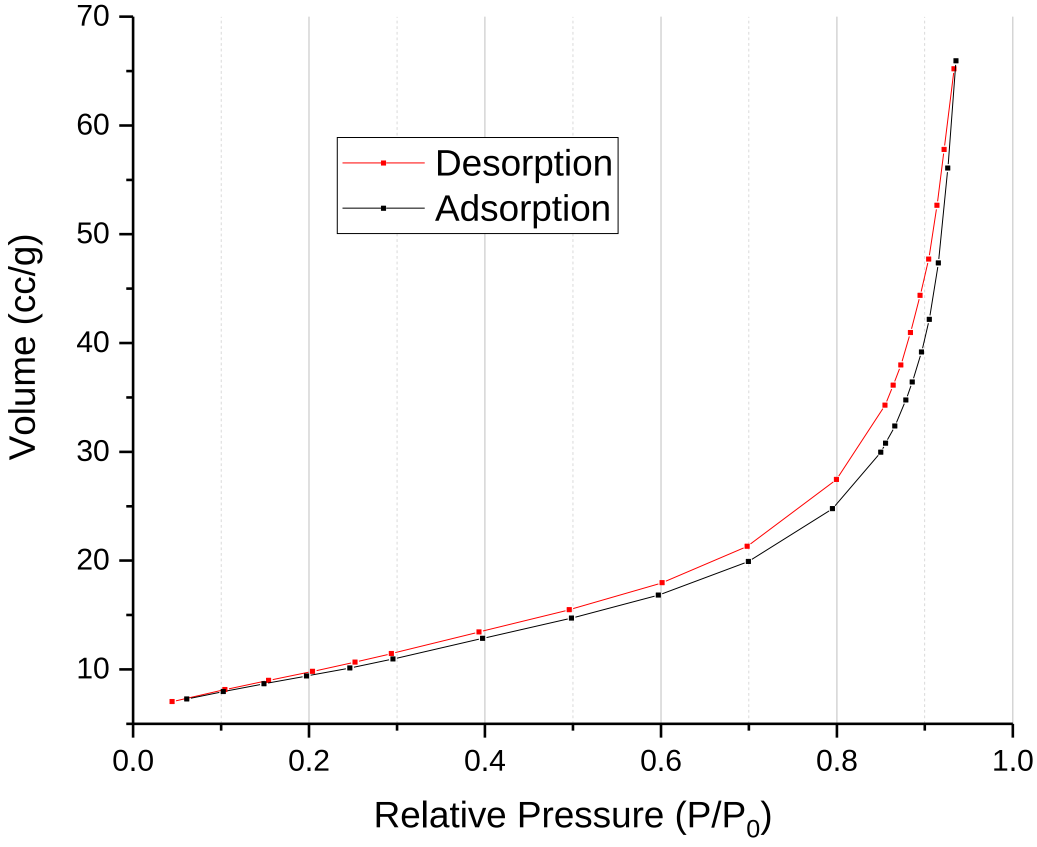

Figure S27: Nitrogen adsorption-desorption isotherm for the BNNS-$CoFe_2O_4$ nanocomposite.

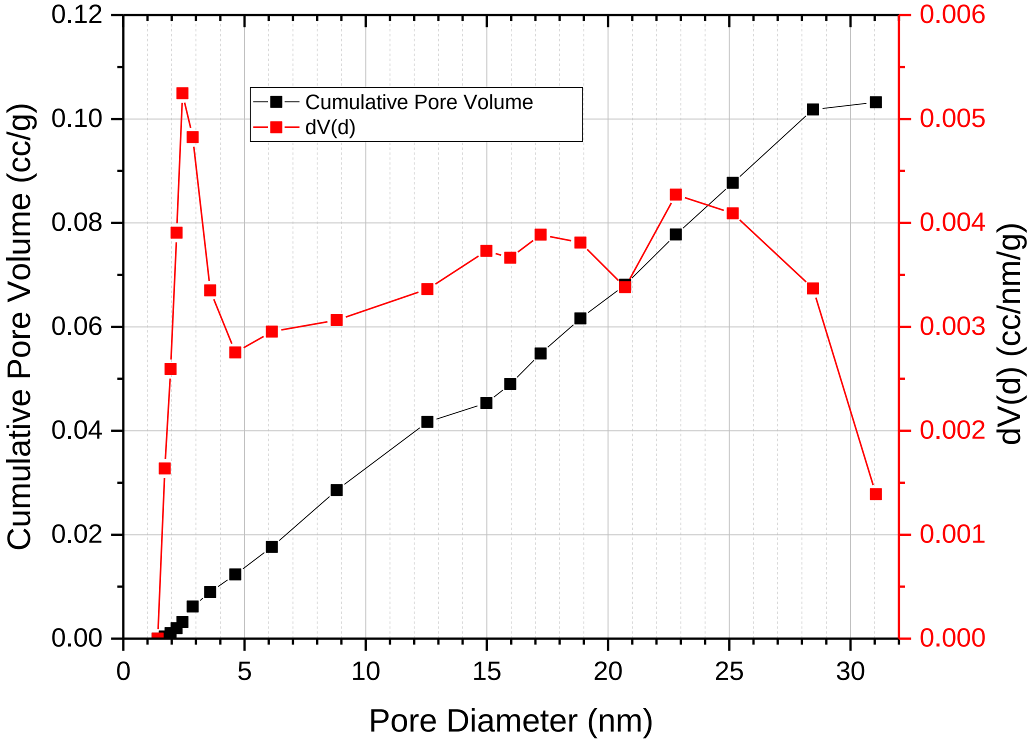

Figure S28: BJH pore size distribution of the BNNS-$CoFe_3O_4$ nanocomposite.

Table S1: Physical characteristics for a membrane of 0.001018 m^2 operating at one bar.

	**BNNS**	**BNNS-Fe_3O_4**	**BNNS-$CoFe_2O_4$**
Mass	40 mg	40 mg	40 mg
Thickness	0.050 mm	0.052 mm	0.053 mm
Flow rate	620 $Lm^{-2}hr^{-1}$	343 $Lm^{-2}hr^{-1}$	492 $Lm^{-2}hr^{-1}$

Table S2: Percentage removal of the MB from 20 ml aliquots for the various membranes and PVDF support

**Cumulative MB volume (ml)**	**BNNS**	**BNNS-Fe_3O_4**	**BNNS-$CoFe_2O_4$**
	% removed	% removed	% removed
20	>99.9	>99.9	>99.9
40	>99.9	>99.9	>99.9
60	99.7	>99.9	>99.9
80	99.5	>99.9	99.0
100	90.4	98.3	70.5
120	72.0	95.9	18.4
140	38.8	94.1	7.95
160	30.1	82.9	
180		63.1	
200		45.0	

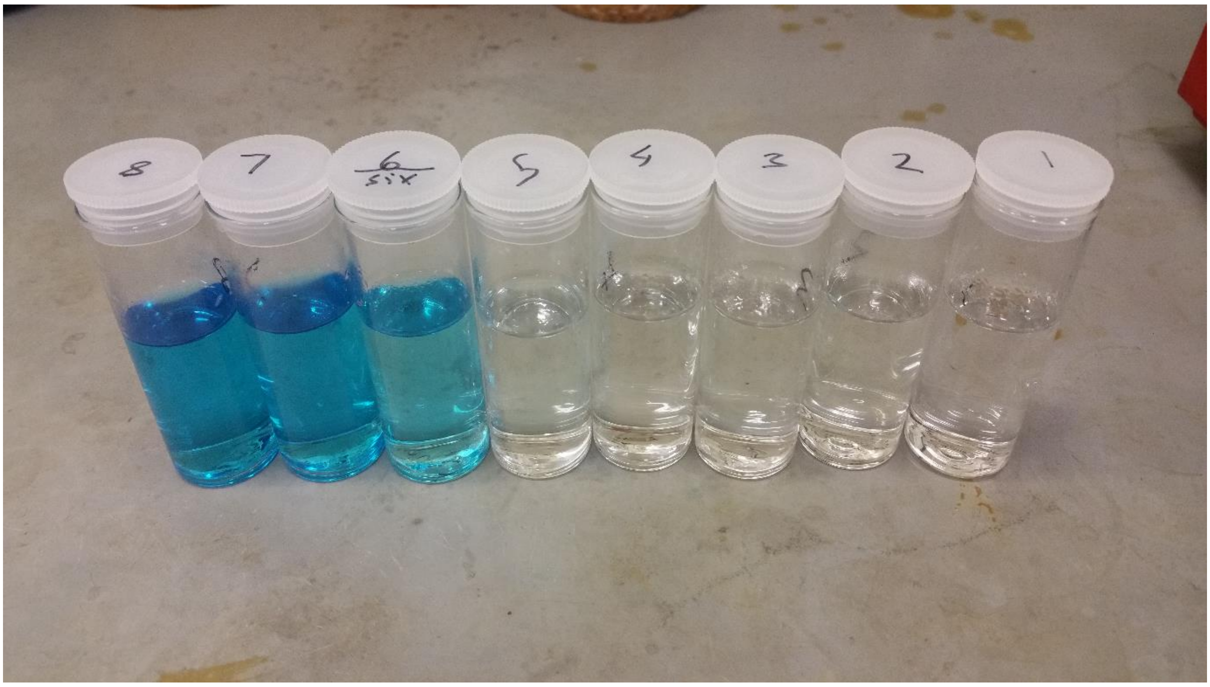

Figure S29: Successive filtrates showing MB after the fifth aliquot for BNNS membrane.

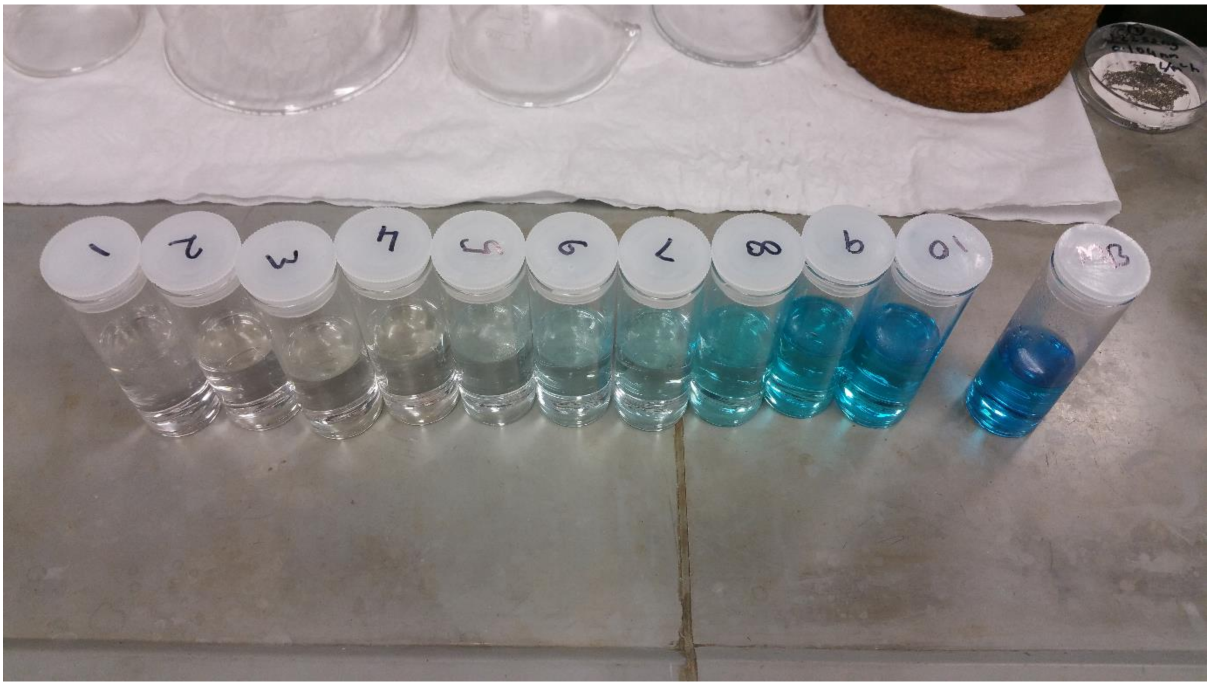

Figure S30: Successive filtrates of the BNNS-Fe3O4 membrane, with the original MB solution on the right.

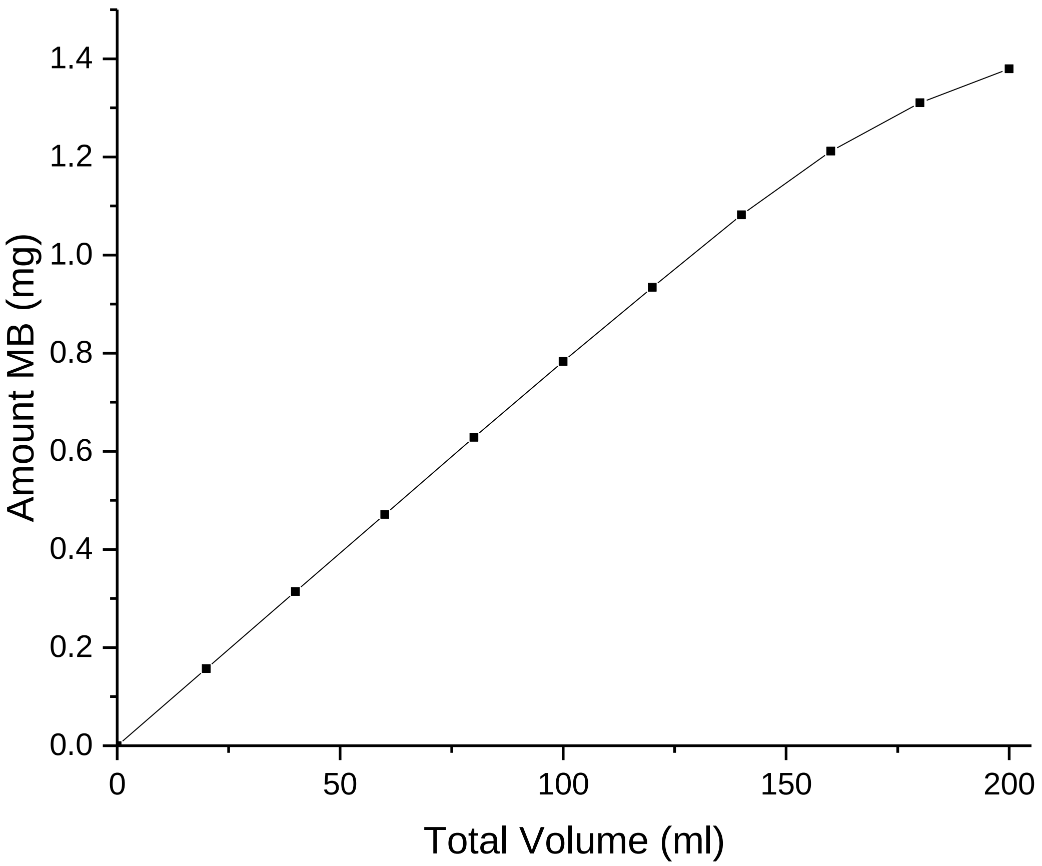

Figure S31: Accumulation of MB on BNNS-Fe_3O_4 membrane until saturation develops.

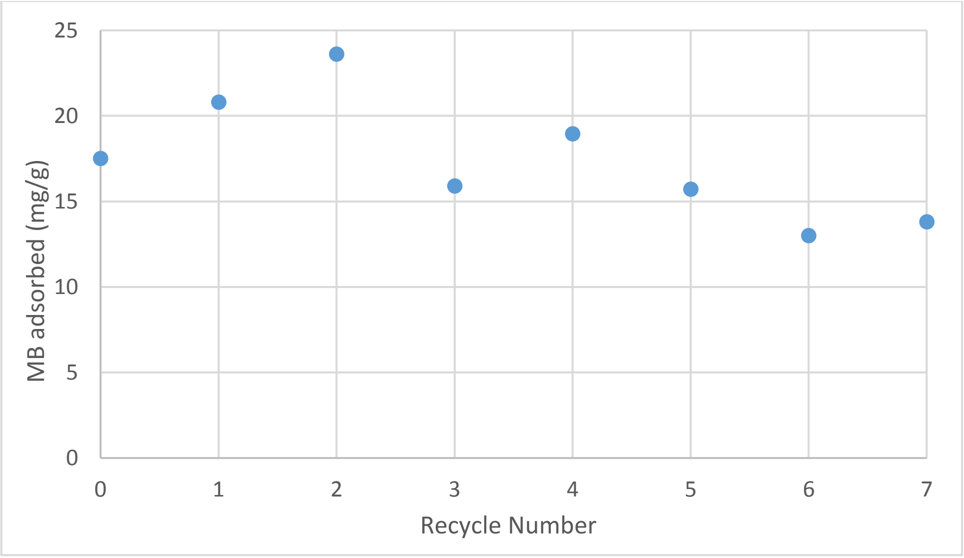

Figure S32: Recycling results for the BNN-$CoFe_2O_4$ nanocomposite

Table S3: Quantification of the MB removed with each recycle, showing the reduction in mass of the membrane material as some is lost in the recycling process.

Recycle Number	BNNS-$CoFe_2O_4$ mass (mg)	Adsorption (mg/g
0	40.0	17.5
1	39.1	20.8
2	38.3	23.6
3	37.1	15.9
4	35.8	18.95
5	35.1	15.7
6	34.0	13
7	34.3	13.8

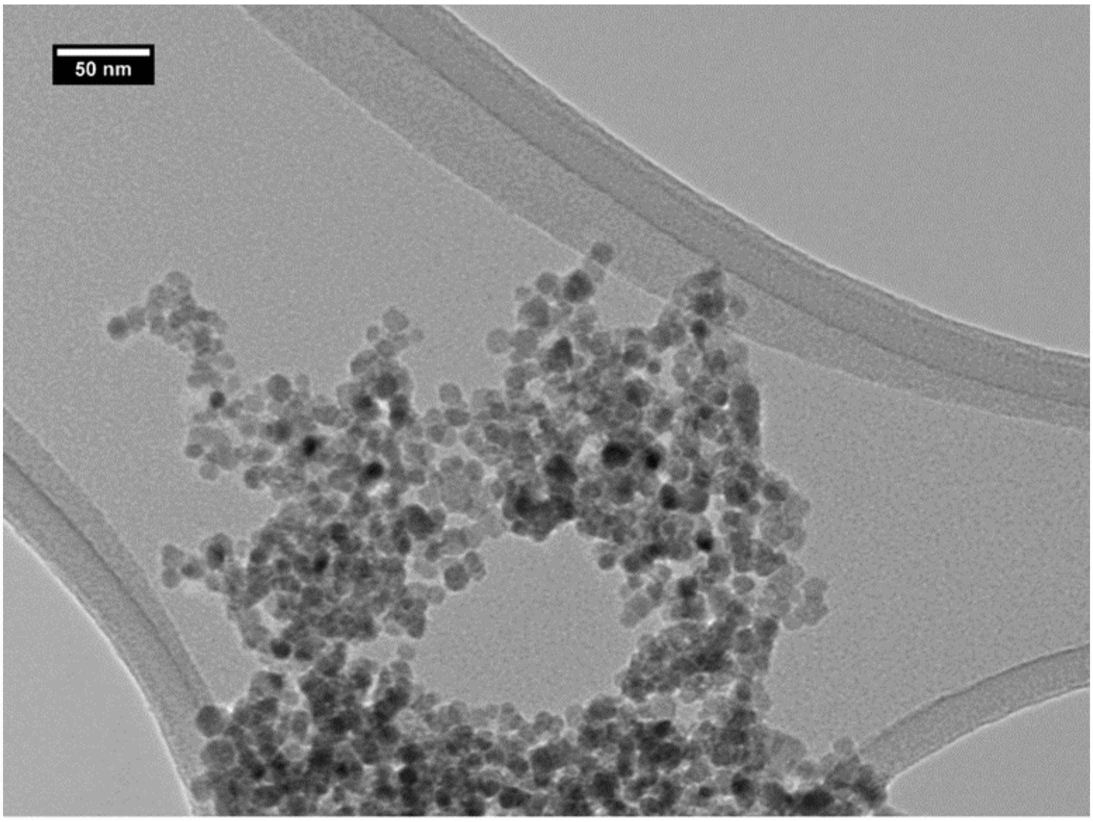

Figure S33: TEM image of the coprecipitated MNPs.

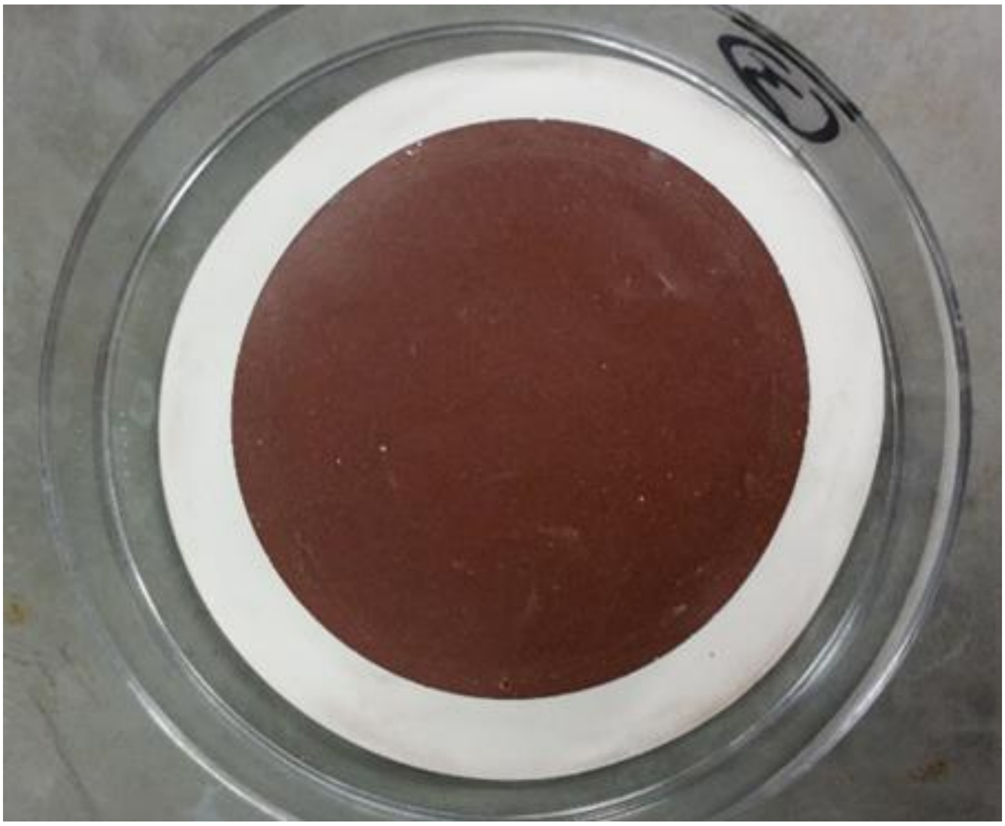

Figure S34: BNNS membrane with captured MNPs (brown) on the PVDF support (white)
